# Worldclim 2.1 versus Worldclim 1.4: Climatic niche and grid resolution affect between‐version mismatches in Habitat Suitability Models predictions across Europe

**DOI:** 10.1002/ece3.8430

**Published:** 2022-02-14

**Authors:** Francesco Cerasoli, Paola D'Alessandro, Maurizio Biondi

**Affiliations:** ^1^ Department of Life, Health and Environmental Sciences – Environmental Sciences Sect. University of L'Aquila L'Aquila Italy

**Keywords:** climate scenarios, climatic niche, grid resolution, Habitat Suitability Models, virtual ecologist, Worldclim

## Abstract

The influence of climate on the distribution of taxa has been extensively investigated in the last two decades through Habitat Suitability Models (HSMs). In this context, the Worldclim database represents an invaluable data source as it provides worldwide climate surfaces for both historical and future time horizons. Thousands of HSMs‐based papers have been published taking advantage of Worldclim 1.4, the first online version of this repository. In 2017, Worldclim 2.1 was released. Here, we evaluated spatially explicit prediction mismatch at continental scale, focusing on Europe, between HSMs fitted using climate surfaces from the two Worldclim versions (between‐version differences). To this aim, we simulated occurrence probability and presence‐absence across Europe of four virtual species (VS) with differing climate‐occurrence relationships. For each VS, we fitted HSMs upon uncorrelated bioclimatic variables derived from each Worldclim version at three grid resolutions. For each factor combination, HSMs attaining sufficient discrimination performance on spatially independent test data were projected across Europe under current conditions and various future scenarios, and importance scores of the single variables were computed. HSMs failed in accurately retrieving the simulated climate‐occurrence relationships for the climate‐tolerant VS and the one occurring under a narrow combination of climatic conditions. Under current climate, noticeable between‐version prediction mismatch emerged across most of Europe for these two VSs, whose simulated suitability mainly depended upon diurnal or yearly variability in temperature; differently, between‐version differences were more clustered toward areas showing extreme values, like mountainous massifs or southern regions, for VSs responding to average temperature and precipitation trends. Under future climate, the chosen emission scenarios and Global Climate Models did not evidently influence between‐version prediction discrepancies, while grid resolution synergistically interacted with VSs' niche characteristics in determining extent of such differences. Our findings could help in re‐evaluating previous biodiversity‐related works relying on geographical predictions from Worldclim‐based HSMs.

## Graphical abstract

Habitat Suitability Models (HSMs) fitted using climate data from the Worldclim repository have been extensively implemented in recent years to tackle questions related to climate and biodiversity. We investigated whether niche characteristics of the target species, grid resolution of climate surfaces, and climate change scenarios selected for model projection, influence prediction mismatch across Europe between HSMs fitted using bioclimatic variables from the first (1.4) and updated (2.1) versions of the Worldclim database. Prediction mismatch is mainly related to the particular climate‐occurrence relationships characterising the target entity. Grid resolution, rather than climate change scenarios, interacts with these latter in determining prediction mismatch to future time horizons.

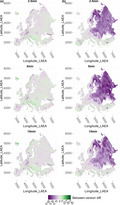

## 1 INTRODUCTION

Climate shapes the distribution of organisms at regional‐to‐global scales (Newbold, 2018; Thuiller et al., 2004) as it constrains the fundamental niche of species (Pearman et al., 2008). The need to quantify climate‐occurrence relationships, which characterizes disciplines like macroecology and phylogeography, has boosted research efforts on predicting the response of species to climate across space and time (Bellard et al., 2013; Nogués‐Bravo et al., 2010).

The spread of Habitat Suitability Models (HSMs) during the last two decades has further expanded the toolbox aiding researchers in tackling questions related to climate and biodiversity (Guisan et al., 2017). Correlative HSMs, also commonly named Species Distribution Models (SDMs) or Ecological Niche Models (ENMs), estimate the relationships between the target biological entity (e.g. species or populations) and a set of abiotic and/or biotic variables (predictors), based on data representing presence, presence‐absence or presence‐background localities (Elith & Leathwick, 2009). Once estimated, such relationships are usually projected onto the geographical space, either limiting predictions to the calibration area (interpolation) or also predicting into new regions and past/future time horizons (extrapolation). HSMs rely upon some fundamental assumptions, notably species‐environment equilibrium and appropriate coverage of the species' realized niche within calibration data (Araújo & Rahbek, 2006; Cerasoli et al., 2021), which question their applicability in situations like recent range expansion by alien species or rapid niche shifts (Pearman et al., 2008). Nonetheless, thoughtfully implemented HSMs represent a powerful tool to investigate the influence of climate on species' range dynamics (Cerasoli et al., 2020; Iannella et al., 2017, 2020; Vega et al., 2010).

To this aim, availability of gridded spatial layers representing climatic conditions across the study area (i.e. climate surfaces) is essential. Among the various sources of climate data, the Worldclim database has represented a breakthrough as it has provided ecological modellers with worldwide climate surfaces at various grid resolutions (from 30 arc‐seconds to 10 arc‐minutes) for “current” conditions (averaging between 1950s and early 2000s) as well as for past (late Pleistocene to middle Holocene) and future (up to 2100) time horizons. The first version of this database was released in 2005 (Hijmans et al., 2005) and successively stabilized in the Worldclim 1.4 online repository, including climate surfaces derived from interpolation of monthly precipitation and temperature data recorded by thousands of meteorological stations around the globe (excluding Antarctica). These baseline climate surfaces were used in the following years to downscale the climate projections to past and future time horizons performed within the Coupled Model Intercomparison Project Phase 5 (CMIP5) of the Working Group on Coupled Modeling. Worldclim was updated in 2017 to version 2.1 (Fick & Hijmans, 2017), which benefited from data recorded by a higher number of meteorological stations, particularly at high latitudes and elevations, and included climate surfaces representing solar radiation, windspeed and vapour pressure in addition to the ones available in Worldclim 1.4. Subsequently, baseline climate surfaces from Worldclim 2.1 were used to downscale the projections to future alternative scenarios derived within the Coupled Model Intercomparison Project Phase 6 (CMIP6). While CMIP5 future scenarios comprised four Representative Concentration Pathways (RCPs) depicting different trajectories of greenhouse gas emissions resulting in increased radiative forcing compared to current values (Moss et al., 2010), CMIP6 extended the range of alternative scenarios by coupling RCPs with a newly developed framework of Shared Socio‐Economic Pathways (SSPs), focused on possible socioeconomic developments influencing adaptation and mitigation policies (Riahi et al., 2017). The stabilized version of the Worldclim 2.1 repository, including current and future climate surfaces, was released in January 2020.

The Worldclim database has been vastly implemented in Habitat Suitability (HS) Modeling to estimate climate influences on the realized niche of species and predict possible distributional shifts driven by climate change: as an example, a simple query of “Worldclim” + “SDMs” (SDMs being the most common labeling of HSMs projected onto the geographical space) in Google Scholar (performed on 27 May 2021) delivered 3550 results, 2660 of which published up to 2019 (i.e. before Worldclim 2.1 was released). Source of climate data is an important driver of uncertainty in HSMs' predictions, according to papers contrasting models fitted using various global climate datasets (Baker et al., 2016; Morales‐Barbero & Vega‐Álvarez, 2019; Watling et al., 2014) or global datasets versus regional ones (Jiménez‐Valverde et al., 2021) as well as versus fine‐scale predictors derived from remote sensing or *in situ* measurements (Deblauwe et al., 2016; Lembrechts et al., 2019). Nonetheless, to the best of our knowledge, no research has so far investigated geographical discrepancies in predictions from climate‐based HSMs fitted using Worldclim 2.1 versus Worldclim 1.4: assessing the geographical arrangement and magnitude of such differences would greatly help to critically evaluate results from the huge number of HSMs‐based studies conducted using Worldclim 1.4 in the light of the recent advances in climate modeling. To fill this gap of information, we analyzed spatially explicit prediction mismatch between HSMs fitted using bioclimatic variables from the two Worldclim versions at the continental scale, focusing on Europe. Moreover, as source of predictors represents one of many factors contributing to uncertainty in model predictions (Connor et al., 2018; Lobo & Tognelli, 2011; Saupe et al., 2012), we also investigated if grid resolution of climate surfaces, niche characteristics of the target entity, and the future scenario considered for HSMs' projection may significantly affect prediction mismatch. To this aim, differently from most of previous research pinpointing the effects of climate data sources on HSMs fitted using occurrences of real‐world species, we implemented the so‐called “virtual ecologist approach” (Zurell et al., 2010). The main advantage of this approach in HSMs‐related studies is the possibility of simulating the environment‐occurrence relationships one wants to investigate, the equilibrium or disequilibrium of the virtual species with the environment, the sampling protocol and possible biases therein: *a priori* control of these conditions permits the evaluation of their influence, and that of various modeling choices, upon HSMs' outcomes with lower risk of drawing conclusions affected by confounding factors (Meynard et al., 2019).

Here, we first simulated occurrence probability across Europe of four virtual species differing in climate‐occurrence relationships and niche breadth. Then, for each virtual species, we fitted HSMs for numerous artificial presence‐absence datasets using climate surfaces from Worldclim 1.4 and Worldclim 2.1 at three grid resolutions; the obtained models were subsequently projected to the European extent under current climate and various future scenarios. Finally, we assessed overall correlation and spatially explicit mismatch between predictions of HSMs obtained using the two Worldclim versions, along with correspondence between model predictions and simulated occurrence probabilities, evaluating possible effects of grid resolution, peculiarities of the simulated species and alternative projection scenarios.

## 2 METHODS

**FIGURE 1:**
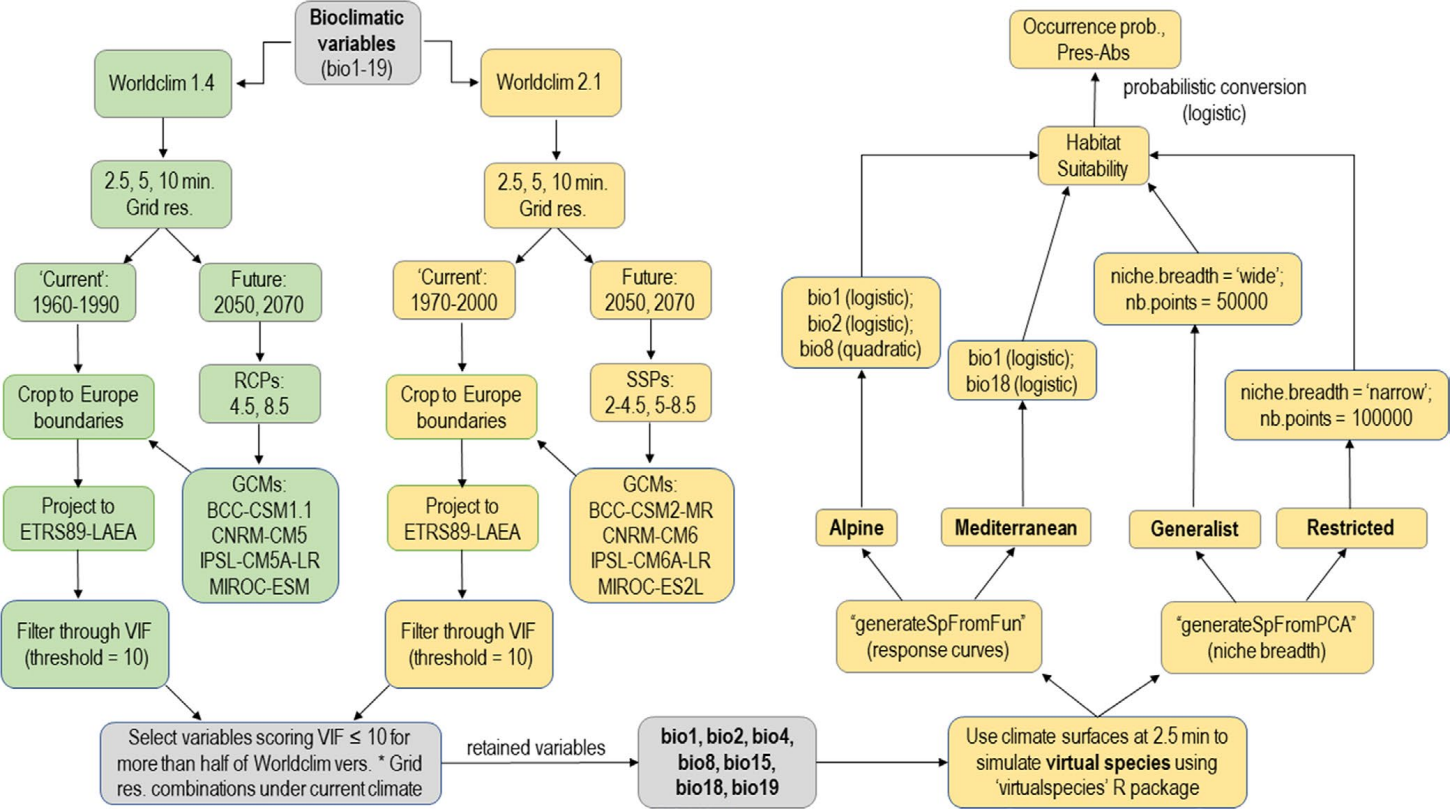
Flowchart resuming the considered combinations of climate surfaces (Worldclim versions, grid resolutions, future scenarios), and the steps leading from variable processing and selection to the simulation of occurrence probability and presence‐absence patterns of the four virtual species. Green boxes show the steps involving climate surfaces derived from Worldclim 1.4, while golden boxes show the steps involving climate surfaces derived from Worldclim 2.1. Grid res. = resolution (arc‐minutes) of the gridded climate surfaces; RCPs = Representative Concentration Pathways; SSPs = Shared Socio‐Economic Pathways; GCMs = Global Climate Models; VIF = stepwise Variance Inflation Factor analysis; nb.points = number of background points on which PCA is performed

**FIGURE 2:**
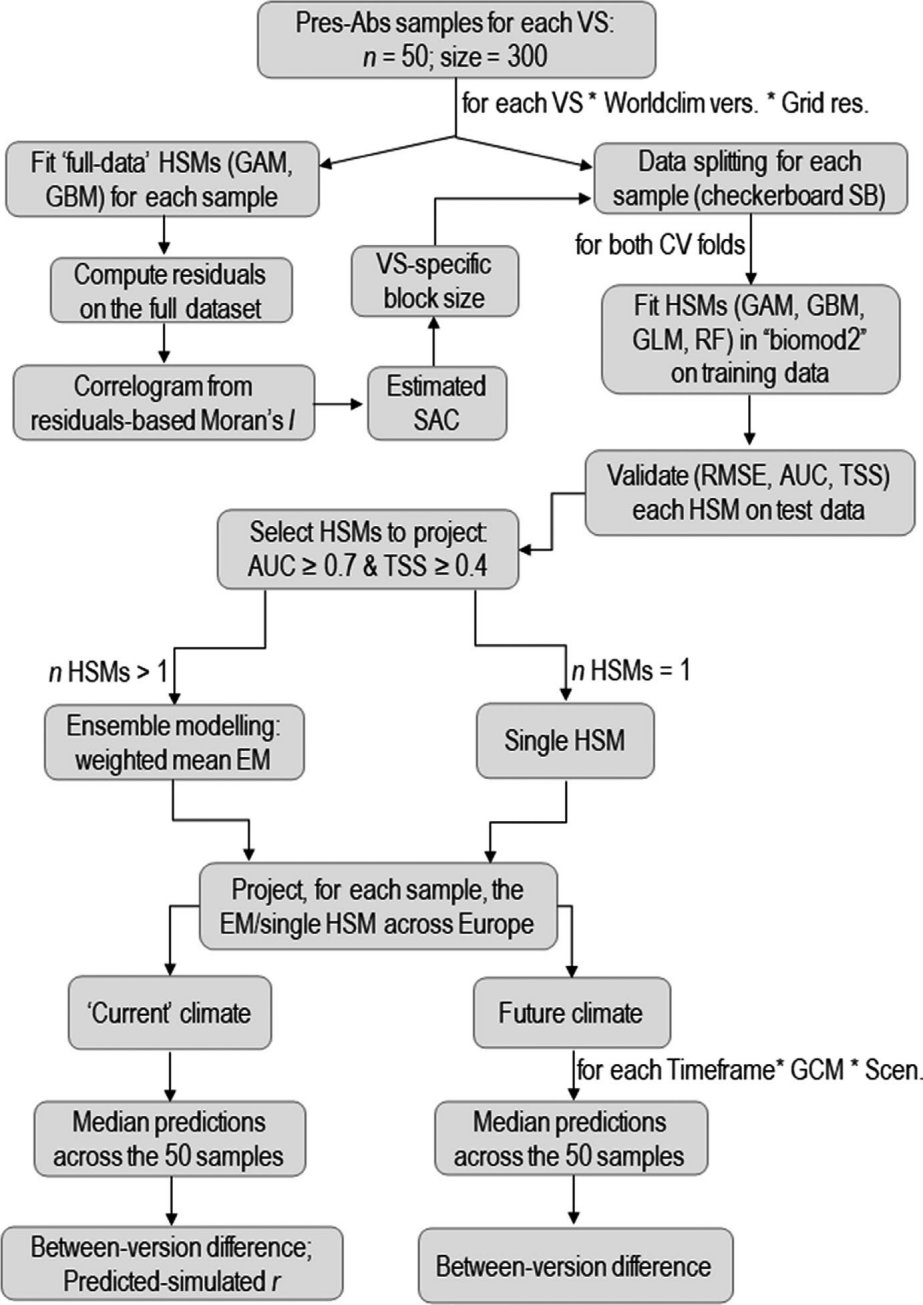
Flowchart resuming the algorithmic workflow implemented to analyse, for each virtual species, between‐version differences in Worldclim‐based HSMs' predictions. VS = virtual species; size = number of records for each sample; Worldclim vers. = version of the Worldclim database (1.4 or 2.1); Grid res. = resolution (arc‐minutes) of the gridded climate surfaces; Moran's *I* = Moran's index; SAC = Spatial Autocorrelation range; checkerboard SB = checkerboard spatial blocking; CV = cross‐validation; HSM = Habitat Suitability Model; EM = Ensemble Model; GCMs = Global Climate Models; Scen. = emission scenario

A schematic representation of the steps leading from the collection, processing, and selection of the climate surfaces to the generation of the presence‐absence datasets for each virtual species is provided in Figure 1, while a similar flowchart is reported in Figure 2 to summarize the steps taken to fit, evaluate, select, and project the HSMs. Details about the entire modeling workflow are provided in the sections below.

### 2.1 Bioclimatic variables

Raster layers of worldwide climate surfaces representing 19 temperature‐ and precipitation‐related bioclimatic variables were downloaded from the Worldclim online repository (https://www.worldclim.org/data/index.html) for both Worldclim 1.4 (Hijmans et al., 2005) and Worldclim 2.1 (Fick & Hijmans, 2017), each at three grid resolutions: 2.5, 5, and 10 arc‐minutes (hereafter 2.5, 5, and 10 min), respectively, corresponding to pixels whose sides roughly measure 5, 10, and 20 km at the equator. Worldclim climate surfaces referring to “current” conditions derive from interpolation of monthly meteorological data averaged across 30 years (1960–1990 in Worldclim 1.4, 1970–2000 in Worldclim 2.1). Climate surfaces representing possible future conditions were downloaded, at 2.5, 5, and 10 min for both Worldclim versions, choosing two alternative hypotheses about global policies characterizing the next decades: the former, represented by the RCP4.5 scenario in Worldclim 1.4 (hereafter Wclim1.4) and by the SSP2‐4.5 one in Worldclim 2.1 (hereafter Wclim2.1; Riahi et al., 2017; Thomson et al., 2011), assumes some degree of international cooperation to limit greenhouse gas emissions and enhance exploitation of renewable energy sources, leading to a stabilized radiative forcing of 4.5 W m^−2^ in 2100; the latter, represented by RCP8.5 in Wclim1.4 and by SSP5‐8.5 in Wclim2.1 (Riahi et al., 2011, 2017), instead assumes increasing emissions from continued exploitation of fossil fuels, limited technological advancement and almost no coordinate efforts to mitigate climate change, resulting in non‐stabilized radiative forcing of at least 8.5 W m^−2^ in 2100. For the sake of simplicity, hereafter we will refer to these two alternative emission scenarios as “Scen4.5” and “Scen8.5,” respectively, for both Worldclim versions. For each scenario, we considered two future timeframes, 2050 and 2070, respectively corresponding to average projections across 2041–2060 and 2061–2080. Previous works (Garcia et al., 2012; Porfirio et al., 2014; Stralberg et al., 2015) showed that the specific Global Climate Models (GCMs) used to project the climate‐occurrence relationships estimated under present conditions may noticeably affect predicted distributional shifts. To account for the possible effect of the choice of GCMs on prediction mismatch between Wclim1.4‐based HSMs and Wclim2.1‐based ones, we downloaded, for each grid resolution * scenario * timeframe combination, climate surfaces derived from four GCMs: for Wclim1.4 we chose BCC‐CSM1.1, CNRM‐CM5, IPSL‐CM5A‐LR, and MIROC‐ESM; for Wclim2.1 we selected updated GCMs from the same modeling groups, namely BCC‐CSM2‐MR, CNRM‐CM6, IPSL‐CM6A‐LR, and MIROC‐ESL2. Hereafter, regardless of the considered Worldclim version, we will refer to these GCMs simply according to the corresponding modeling group: the Chinese BCC (Beijing Climate Center); the French CNRM (Centre National de Recherches Météorologiques) and IPSL (Institute Pierre‐Simon Laplace); the Japanese MIROC (Model for Interdisciplinary Research On Climate).

Using the “raster” (Hijmans, 2020) R (R Core Team, 2020) package, current and future climate surfaces were cropped to the European geographical boundaries (spatial extent: Longitude 24.55°O–61.68°E, Latitude 34.56°N–73.43°N). All the climate surfaces were then projected from the original WGS84 geographic coordinate system to the ETRS89‐LAEA projected one to get equally sized raster cells across the European extent.

As multicollinearity among predictors may substantially affect HSMs, biasing the fitted environment‐occurrence relationships and the estimated importance scores of the single predictors (Dormann et al., 2013), we used the stepwise Variance Inflation Factor (VIF) analysis implemented in the “usdm” R package (Naimi et al., 2014) to filter the initial set of nineteen bioclimatic variables: the variables not exceeding VIF = 10 (Guisan et al., 2006; Werkowska et al., 2017) for more than half of the Worldclim version * grid resolution combinations were retained to simulate the virtual species and fit the HSMs.

Values of the selected temperature‐related variables from Wclim1.4 were divided by 10, because in Wclim1.4 temperature is reported as degree celsius * 10 while in Wclim2.1 it is reported as “plain” degree celsius. Then, climate surfaces from Wclim1.4 were resampled through bilinear interpolation to match dimensions of Wclim2.1‐derived ones, thus permitting pixel‐by‐pixel comparison across Europe of predictions from HSMs fitted using climate surfaces from the two Worldclim versions. Finally, for each retained variable, we assessed correlation between climate surfaces from the two versions (hereafter between‐version *r*) in two ways. First, computing pairwise Pearson's *r* on sets of points drawn through regular random sampling across Europe, comparing the “original” layers (i.e., prior to the resampling of Wclim1.4‐derived ones) from both Worldclim versions, as well as the resampled Wclim1.4‐derived layers with the Wclim2.1‐derived ones. Second, computing local Pearson's *r* across the entire European extent through a focal neighborhood analysis contrasting the resampled Wclim1.4‐derived layers with the Wclim2.1‐derived ones.

### 2.2 Virtual species

Taking advantage of the “virtualspecies” R package (Leroy et al., 2016), we simulated HS across Europe of four virtual species (hereafter VS). One of the VSs represents a climate‐tolerant species (“Generalist”), while the remaining three correspond to different typologies of climate‐constrained species: (i) a cold‐adapted species (“Alpine”), whose distribution is limited to mountainous areas in southern Europe while being wider at high latitudes; (ii) a species preferring warm and dry conditions, thus mainly occurring within the Mediterranean European regions (“Mediterranean”); (iii) a species adapted to a poorly represented combination of climatic conditions, thus occurring in narrow and geographically sparse areas (“Restricted”).

**FIGURE 3:**
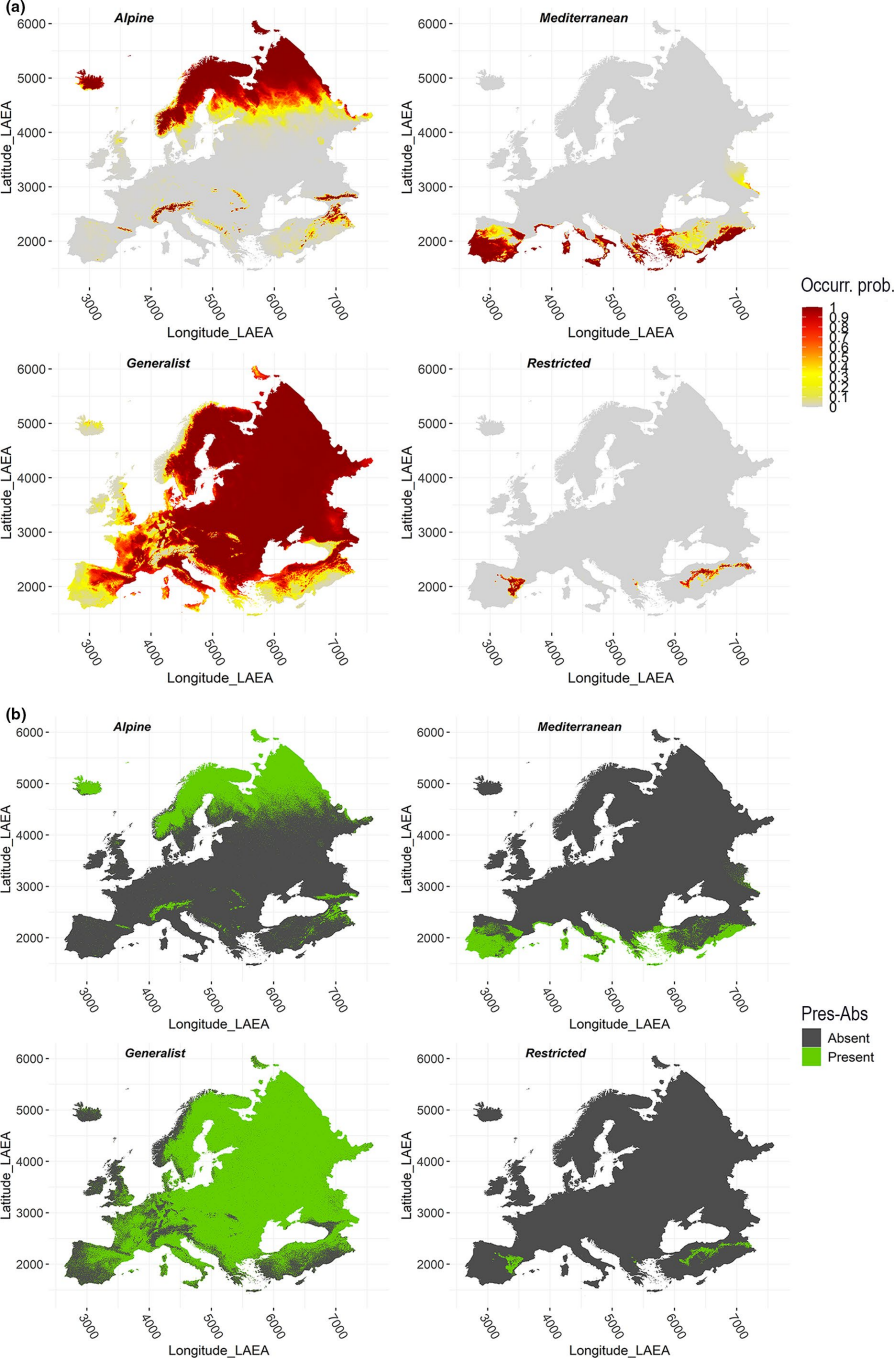
(a) Occurrence probability and (b) presence‐absence maps, for each VS, resulting from the probabilistic conversion of the simulated HS

HS for the Alpine and Mediterranean VSs was simulated through customized response curves to some of the retained variables, while HS for the Generalist and Restricted VSs was modelled as a function of niche breadth within a two‐dimensional gridded space resulting from a preliminary Principal Component Analysis (PCA) applied to the retained variables. Density of occurrence of the Generalist and Restricted VSs within the PCA‐derived 2D space was estimated by means of the “ecospat” R package (Di Cola et al., 2017). Details about the functions used to simulate responses of the Alpine and Mediterranean VSs to the selected variables, as well as about the PCA performed for the Generalist and Restricted VSs, are provided in Appendix Note S1. The resulting HS maps were visually checked to ensure they matched the climate‐occurrence relationships we intended to simulate (Meynard et al., 2019). Then, a probabilistic approach was used to convert, for each pixel, the HS value into a corresponding occurrence probability value (Figure 3a) and then into a presence‐absence (hereafter Pres‐Abs) record (Figure 3b) based on a logistic function: relying on a probabilistic approach rather than on a threshold‐based one permits to avoid abrupt changes in the relationship between HS and the resulting occurrence probability (i.e. threshold‐like responses), which are rare in real‐world circumstances (Meynard & Kaplan, 2013). Within the logistic function used to convert occurrence probability into Pres‐Abs records, lower *α* (slope) and *β* (inflexion point) absolute values were set for the Generalist (*α* = −0.01; *β* = 0.4) than for the other VSs to obtain a considerably wider extent of occurrence for the former, thus simulating a species adapted to a broad range of climatic conditions. The remaining VSs shared the same slope value (*α* = −0.05), while the inflexion point was adjusted in a species‐specific manner (Alpine: *β* = 0.6; Mediterranean: *β* = 0.7; Restricted: *β* = 0.8), so as to get a relatively wide distribution for the Alpine VS at high latitudes and across the main European mountainous massifs, a somewhat narrower distribution for the Mediterranean one, mainly occurring in southern coastal areas except for some inland regions of Iberia, southern Italy, Greece, and Turkey, and an even narrower distribution for the Restricted VS (Figure 3b). The “virtualspecies” package also allows to limit the actual distribution of VSs to a user‐defined portion of the occurrence area resulting from the conversion of the simulated HS to Pres‐Abs patterns, thus mimicking species being not in equilibrium with climate (Leroy et al., 2016). Here, following Meynard et al. (2019) who suggested that studies based on VSs should keep as simple as possible the factors not under investigation, we simulated VSs being in equilibrium with climate.

For all the VSs, the baseline climate surfaces used to simulate HS were those from Wclim2.1 at 2.5 min. The rationale behind this choice was twofold. First, for the Alpine and Mediterranean species we could also have made a separate HS simulation for each Worldclim version * grid resolution combination as suitable conditions for these VSs were related to specific value ranges of selected variables via customized response functions, thus being independent of the particular climate surfaces used; instead, doing the same for the Generalist and Restricted species would have produced different climate‐occurrence relationships depending on the considered Worldclim version * grid resolution combination, because HS for these VSs was related to their overall niche breadth in the PCA‐derived 2D space summarizing a specific set of climate surfaces. Second, simulating HS using the climate surfaces at 2.5 min as reference layers for all the VSs permitted to also assess to what extent HSMs fitted using coarser climate surfaces were able to correctly retrieve climate‐occurrence relationships acting at a finer scale (Connor et al., 2018).

For each VS, 50 samples, each comprising 300 Pres‐Abs points, were drawn from the simulated Pres‐Abs maps. The effect of sample size on HSMs' predictive performance has been variously investigated in recent years, with some studies suggesting that good accuracy may be attained with at least 25–30 training presences (van Proosdij et al., 2016; Wisz et al., 2008) while others claiming for the need to collect more presence records (Hanberry et al., 2012; Santini et al., 2021). The sample size we chose aimed at balancing the need for sufficient calibration data with that of making the results of our simulations comparable to those researchers may get when fitting Worldclim‐based HSMs to datasets collected for real‐world species, which often comprise <50 presence points (Santini et al., 2021). Except for the Generalist VS, sampling was limited to a buffer area (120 km for Alpine and Mediterranean VSs, 60 km for the Restricted one) around a randomly chosen sample of 500 presence pixels. Indeed, if Pres‐Abs records for the three climate‐constrained VSs had been sampled from the entire European extent, the risk of generating datasets with very low sample prevalence would have been high, which in turn would have biased the estimation of HSMs' accuracy (Jiménez‐Valverde, 2021). As discrimination scores on test data were later used to select the HSMs to be passed thourgh the projection phase (see *Model fitting and projection*), it was essential that such estimates were as unbiased as possible. On the other hand, this choice presumably introduced a certain degree of extrapolation in the HSMs' projection phase for the climate‐constrained VSs, particularly for the Restricted one whose occurrence area was notably narrower (and thus likely covered fewer combinations of climatic conditions) than for the others. However, this was beneficial to the aim of our research, as we wanted to investigate between‐version differences in Worldclim‐based HSMs not only for interpolation tasks but also when the models have to predict outside calibration areas, given that this latter condition characterizes most of HSMs' practical applications.

Finally, we simulated unperfect detection during the “virtual sampling”, here again in order to make modeling conditions on our VSs relatively similar to those researchers working on real‐world datasets (e.g., cryptic target species, lowly experienced observers) usually face: specifically, the “detection.probability” value in the “convertToPA” function from the “virtualspecies” package was set to 0.75 for the Alpine, Mediterranean, and Generalist VSs, and to 0.9 for the Restricted one. The higher detection probability chosen for the Restricted VS aimed at making its Pres‐Abs datasets containing few false absences; indeed, a high number of false absences would have further decreased its sample prevalence, which was likely *a priori* lower than that of the other VSs given its limited extent of occurrence.

### 2.3 Model fitting and projection

First, for each combination of VS * retained variable * grid resolution, we computed between‐version pairwise *r* on each Pres‐Abs sample to preliminary assess whether diverging input values between the two Worldclim versions could differently influence the climate‐occurrence relationships estimated in the corresponding HSMs.

Then, we investigated for each VS the possible presence of spatial autocorrelation (SAC) affecting the simulated Pres‐Abs data. Indeed, occurrence records and associated environmental data usually show spatial dependence structures (i.e., nearby sites host more similar environmental conditions than distant ones) which, if disregarded when fitting HSMs, frequently lead to biases in the estimation of model parameters and prediction error (Dormann, 2007; Roberts et al., 2017). In particular, if HSMs are validated on randomly withheld data being environmentally close to the training ones due to underlying SAC, estimates of model predictive performance will be inflated (Veloz, 2009). As mentioned above, we needed our estimates of model accuracy to be not biased as they were later used for model selection. Thus, we implemented a cross‐validation scheme which takes advantage of geographically designed blocks to split train and test data accounting for SAC. To this aim, we fitted “full‐data” HSMs (i.e., using all the Pres‐Abs points from each sample) for each VS * Worldclim version * grid resolution combination using Generalized Additive Models (GAM; Guisan et al., 2002) and Generalized Boosted Regression Models (GBM), also known as Boosted Regression Trees (Elith et al., 2008), respectively, implemented through the “gam” (Hastie, 2019) and “gbm” (Greenwell et al., 2020) R packages. GAM and GBM emerged in previous studies among the best performing algorithms in terms of both interpolation and extrapolation accuracy (Heikkinen et al., 2012; Qiao et al., 2019). Residuals of predictions from these “full‐data” HSMs were then used to derive, through the “ncf” (Bjornstad, 2020) R package, correlograms showing variations in residuals‐based Moran's index (*I*) at increasing inter‐point distance: the distance after which Moran's *I* approaches 0 indicates the SAC range (Roberts et al., 2017). In order to improve the spatial independence of test data from training ones, the size of spatial blocks should be greater than the SAC range (Roberts et al., 2017). Thus, a specific block size was selected for each VS by increasing the SAC range visually estimated from the corresponding correlograms by 100 km. Then, we used the “blockCV” (Valavi et al., 2019) R package to design spatial blocks of the chosen block size: for each VS * Worldclim version * grid resolution combination, the obtained blocks were grouped in two folds through a checkerboard blocks‐to‐fold assignment, which ensures a more equal distribution of the environmental space across folds compared to contiguous or random blocking, avoiding excessive extrapolation when HSMs predict on test folds (Roberts et al., 2017). Successively, we fitted new HSMs upon training data from each of the two obtained folds, taking advantage of the “biomod2” (Thuiller et al., 2020) R platform and using four algorithms: in addition to GAM and GBM, we also used Generalized Linear Models (Guisan et al., 2002) and Random Forests (RF) (Breiman, 2001), two of the most widely implemented algorithms in HS Modeling. This way, a total of 2400 HSMs were fitted for each VS (2 Worldclim versions * 3 grid resolutions * 50 Pres‐Abs samples * 4 algorithms * 2 blocks‐to‐folds assignments). Details about model parameterization are provided in Appendix Note S2. Although fine‐tuning of the single algorithms based on the specific climatic niche, simulated distribution and “virtual sampling” of each VS could have led to a better calibration of some HSMs (Hao et al., 2020), we chose the “biomod2” ensemble modeling platform, with commonly used parameterization settings for all the VSs, because we intended to investigate the extent and determinants of between‐version discrepancies in Worldclim‐based HSMs under modeling protocols which have been vastly implemented in the ecological literature in the past two decades (Hao et al., 2019).

The predictive performance of HSMs on test folds was assessed through: (i) Root Mean Squared Error (RMSE), measuring the squared mean distance between the predicted HS and the Pres‐Abs values; (ii) Area Under the Curve (AUC) of the Receiver Operating Characteristic (ROC) plot, contrasting sensitivity (i.e., true positive rate) versus 1‐specificity (i.e., false positive rate) along a continuous gradient of binarization thresholds (Fielding & Bell, 1997); (iii) True Skill Statistic (TSS = sensitivity + specificity − 1) (Allouche et al., 2006), computed upon single‐threshold‐based binarized predictions. Only the HSMs attaining at least AUC = 0.7 and TSS = 0.4 when validated on the test data were retained for the subsequent phases: we chose these “relaxed” thresholds to feed the HSMs' projection process with a large enough number of better‐than‐random models for all the factor (i.e., VS, Worldclim version, grid resolution) combinations. If two or more HSMs were retained for a sample, a weighted ensemble model (hereafter EM_wmean_) was built using the “wmean” algorithm in biomod2, assigning weights to the single HSMs based on the corresponding attained AUC score (Marmion et al., 2009). Standardized importance scores (hereafter Std_Imp) of input predictors were computed for the single retained HSM (hereafter HSM_unique_) or for the EM_wmean,_ depending on the considered sample, through the permutation‐based algorithm‐independent procedure implemented in “biomod2” (Thuiller et al., 2009). Then, EM_wmean_ and HSM_unique_ were projected throughout Europe under current climatic conditions as well as under the different future timeframe * GCM * emission scenario combinations, and median predictions across the projected models were computed for each combination. For current projections, we assessed between‐version differences in median predicted HS as well as Pearson's correlation coefficient between median predicted HS and simulated occurrence probability (hereafter predicted‐simulated *r*), while for future projections we only assessed between‐version differences in median predictions.

### 2.4 Importance of modeling factors

We assessed the relative importance of the considered factors upon modeling outcomes fitting RF regression models (Santini et al., 2021), with response variable iteratively set to: (i) Std_Imp of input variables; (ii) predicted‐simulated *r* computed upon 50 samples for each factor combination, with each sample comprising 10,000 randomly drawn pixels; (iii) between‐version differences in median predicted HS under future scenarios, with difference values sampled upon 10,000 randomly drawn pixels for each factor combination. RF models were fitted through the “randomForest” R package (Liaw & Wiener, 2002) upon 1000 trees, with the “mtry” parameter left as default (i.e. $\text{mtry}=\frac{\text{number of predictors}}{3}$). Standardized importance score of each factor was computed by applying the “biomod2” permutation‐based procedure to the fitted RF models.

## 3 RESULTS

### 3.1 Input variables

Based on VIF analysis, seven bioclimatic variables were selected for model fitting: bio1 (annual mean temperature), bio2 (mean diurnal temperature range), bio4 (temperature seasonality), bio8 (mean temperature of wettest quarter), bio15 (precipitation seasonality), bio18 (precipitation of warmest quarter), and bio19 (precipitation of coldest quarter).

Between‐version local Pearson's *r* varied across variables and grid resolutions (Appendix Figure S1a–c). At 2.5 min, values of bio1 were positively correlated within most European regions; correlation maps obtained for bio8, bio18, and bio19 showed wide extents of positive *r* in southern Europe, while scattered areas of negative correlation emerged mainly in central and north‐eastern Europe; for bio2, bio4, and bio15, wide areas of negative correlation emerged across Europe. These trends persisted at coarser grid resolutions, although positively correlated areas were generally wider. Differently, between‐version pairwise correlation computed on the 50 sets of randomly sampled points exceeded *r* = 0.85 for all the variable * grid resolution combinations, without noticeable differences between “original” and resampled Wclim1.4‐derived variables when compared to Wclim2.1‐based ones (Appendix Figure S2). This suggests that the resampling of Wclim1.4‐derived layers, necessary to conduct the subsequent spatially explicit comparisons of model predictions derived from the two Worldclim versions, did not substantially alter cell‐by‐cell values of Wclim1.4 variables. Similar to what appeared from the maps of local Pearson's *r*, bio2 was the variable with the lowest between‐version pairwise correlation.

### 3.2 Virtual species

The Alpine VS was related negatively to bio1 and positively to bio2 via sigmoid curves, while it was related to bio8 through a bell‐shaped curve peaking between 0°C and 5°C (Appendix Figure S3a); the Mediterranean VS was instead positively related to bio1 via a sigmoid curve showing a steep increase after 5°C, and negatively related to bio18 via a threshold‐like function making suitability dramatically decrease after 100 mm (Appendix Figure S3b). The Generalist VS was primarily associated to the positive semiaxis of the first Principal Component (PrinComp1) to which bio4 contributed the most, followed by bio8 and bio18 (Appendix Figure S4a1); differently, the Restricted VS was mainly related to the negative semiaxes of both PrinComp1 and PrinComp2, to which bio2 contributed the most (Appendix Figure S4b1), followed by bio15 and bio1. As expected, density of occurrence within the PCA‐derived 2D climatic space was far wider for the Generalist VS than for the Restricted one (Appendix Figures S4a2 and S4b2). Occurrence probability and Pres‐Abs patterns properly reflected, for all the VSs, the realization into the European geographic space of the climate‐occurrence relationships we intended to simulate (Figure 3a,b). Prevalence of the VSs across Europe was: Alpine = 0.25, Mediterranean = 0.08, Generalist = 0.79, and Restricted = 0.01; median prevalence across the 50 Pres‐Abs samples was relatively close to the prevalence across Europe for the Alpine (~0.4) and Generalist (~0.6) VSs, while it was noticeably higher than this latter for the Mediterranean (~0.36) and Restricted (~0.25) ones (Appendix Figure S5a). Between‐version *r* of the selected variables upon Pres‐Abs points was higher than 0.8 for all the VS * grid resolution combinations, except for bio2 showing median *r* = 0.4 for the Restricted VS (Appendix Figure S5b).

### 3.3 Model fitting and evaluation

The species‐specific block sizes chosen based on the SAC ranges estimated from the correlograms derived from residuals of the “full‐data” HSMs (Appendix Figure S6a–d) were: Alpine = 1600 km; Mediterranean = 600 km; Generalist = 1600 km; Restricted = 400 km. An example of the resulting checkerboard blocking is shown for each VS in Appendix Figure S7a,b.

RMSE computed upon test blocks did not show clear between‐version differences across the VS * grid resolution combinations, although GBM and RF attained slightly lower median RMSE when fitted using Wclim2.1‐derived variables, especially for the Restricted VS (Appendix Figure S8). Median RMSE was higher for the Generalist VS (~0.45) than for the others (~0.40) considering all the algorithms except GAM, which performed poorly (median RMSE = 0.65) for all VSs, suggesting that the used “biomod2” GAM parameterization (“mgcv” algorithm with cross‐validation‐based selection of smoothing parameters) did not lead to a proper calibration of the corresponding HSMs.

While for the Alpine and Mediterranean VSs all the sampling replicates produced at least an HSM exceeding the chosen discrimination thresholds, some replicates with no HSMs entering the projection phase emerged for the remaining VSs (Appendix Table S1), confirming higher difficulty of the HSMs fitted for these latter in retrieving the simulated climate‐occurrence relationships.

### 3.4 Between‐version differences

**FIGURE 4:**
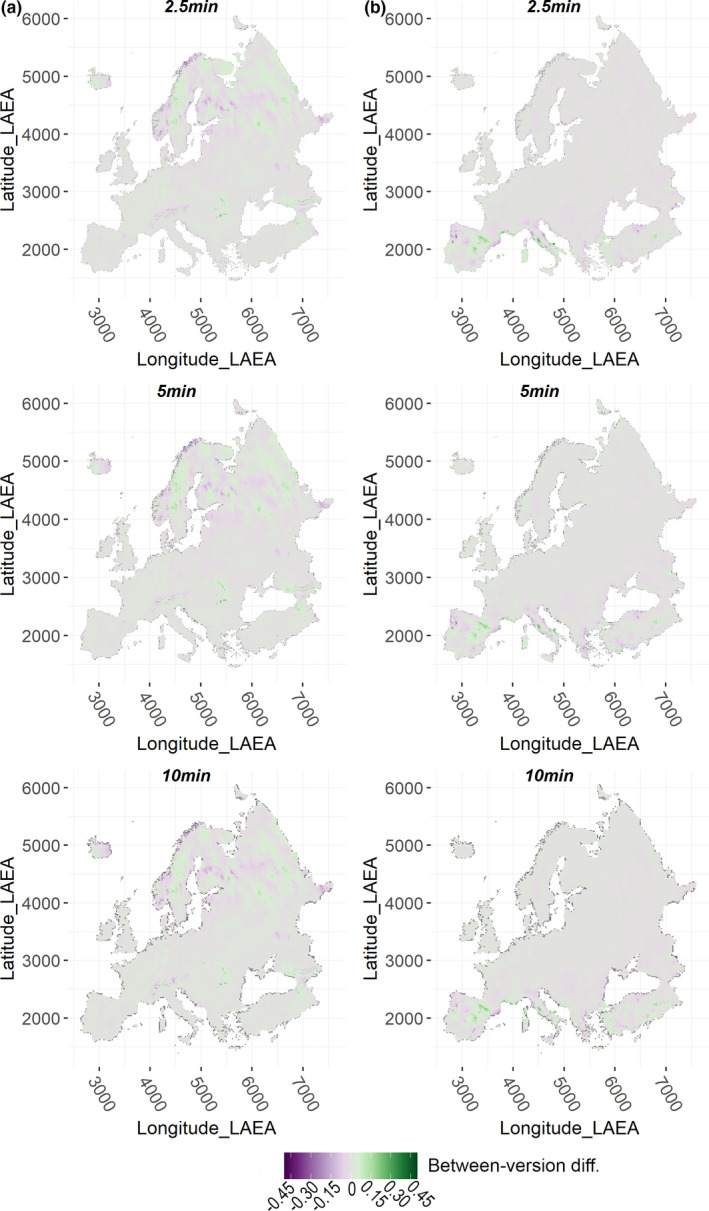
Spatially explicit difference between median Habitat Suitability (HS) predicted by the HSMs fitted using Worldclim 2.1‐based variables (and selected for the projection phase) and median HS predicted by the HSMs fitted using Worldclim 1.4‐based variables (and selected for the projection phase), for the (a) Alpine and (b) Mediterranean VSs

**FIGURE 5:**
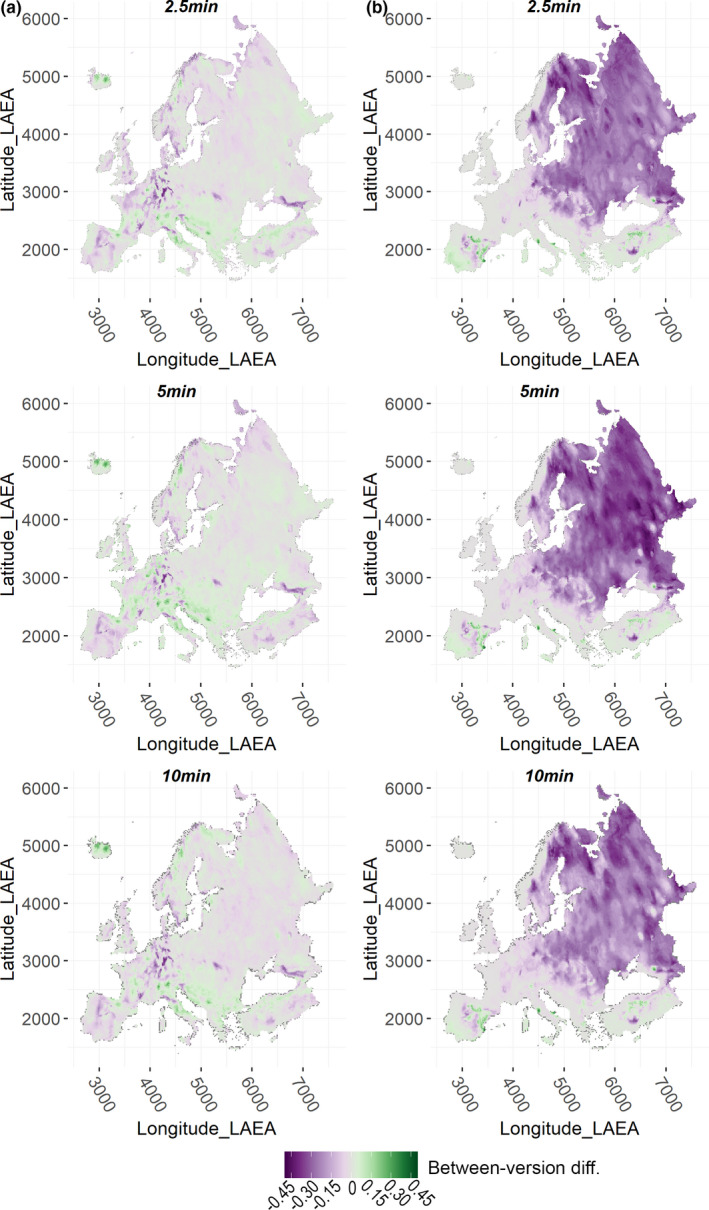
Spatially explicit difference between median Habitat Suitability (HS) predicted by the HSMs fitted using Worldclim 2.1‐based variables (and selected for the projection phase) and median HS predicted by the HSMs fitted using Worldclim 1.4‐based variables (and selected for the projection phase), for the (a) Generalist and (b) Restricted VSs

Between‐version *r* across Europe in median predicted HS was very high, regardless of grid resolution, for the Alpine, Mediterranean and Generalist VSs, while it was somewhat lower for the Restricted one (Appendix Table S2). Looking at spatially explicit prediction discrepancies for the Alpine VS, Wclim2.1‐based projections resulted in higher median HS than Wclim1.4‐based ones across Carpathians, in the central regions of Norway and Sweden, in some areas of northern Finland, north‐eastern European Russia, and north‐eastern Turkey, while the opposite emerged across most of the Alpine arc, in eastern Iceland, south‐western Norway, southern Finland, and some regions of north‐western European Russia (Figure 4a). For the Mediterranean VS, Wclim2.1‐based median HS exceeded Wclim1.4‐based one within eastern inland Iberia, southern French coasts, central Tyrrhennian coasts and southern Adriatic coasts in Italy, as well as in some scattered regions of Anatolia, while the opposite emerged in north‐western Iberia, at the southern Spain‐France border, northern Corse, northern Tyrrhenian and Adriatic Italian coasts, in some territories of Peloponnese and northern Anatolia (Figure 4b). Between‐version differences in median HS involved considerably wider extents for the remaining VSs: Wclim2.1‐based projections for the Generalist VS resulted in lower median HS than Wclim1.4‐based ones for most of Iberia, central‐northern Europe, southern European Russia and Turkey, while the opposite resulted for Iceland, most of the Italian peninsula and the Balkans (Figure 5a); considering the Restricted VS, Wclim2.1‐based median HS was lower than Wclim1.4‐based one across most of eastern Europe, with particularly substantial differences in eastern Scandinavia and European Russia, while more mixed patterns emerged in eastern Iberia and central Anatolia (Figure 5b), the regions showing high simulated occurrence probability for this VS (Figure 3a). These trends were consistent across the three grid resolutions, although between‐version differences were somewhat milder as cell size increased.

Considering standardized importance scores (Std_Imp), HSMs fitted for the Alpine and Mediterranean VSs correctly retrieved as preponderant the predictors upon which HS was simulated (Appendix Figure S9). Indeed, bio1 attained the far highest importance score (median Std_Imp = 75%–80%) for the Alpine VS, followed by bio8 (median Std_Imp = 5%–10%), as well as for the Mediterranean one (median Std_Imp = 55%–60%), here followed by bio18 (median Std_Imp = 15%–20%). Median Std_Imp of bio18 reported for the Mediterranean VS from Wclim2.1‐based HSMs was higher than that resulting from Wclim1.4‐based models across the grid resolutions, suggesting a better characterization of the simulated climate‐occurrence relationships within Wclim2.1‐based HSMs. Differently, bio19 emerged as the preponderant predictor for the Generalist (median Std_Imp = 35%–50%) and Restricted (median Std_Imp = 20%–45%) VSs, with Wclim1.4‐based HSMs overestimating its importance compared to Wclim2.1‐based ones. The fact that the variables mostly contributing to the PCA axes to which these latter VSs were primarily associated (bio4 for the Generalist VS, bio2 for the Restricted one; Appendix Figure S4) obtained far lower Std_Imp than bio19 across all the Worldclim version * grid resolution combinations suggests that the HSMs fitted for these VSs did not properly model their simulated climate‐occurrence relationships.

**FIGURE 6:**
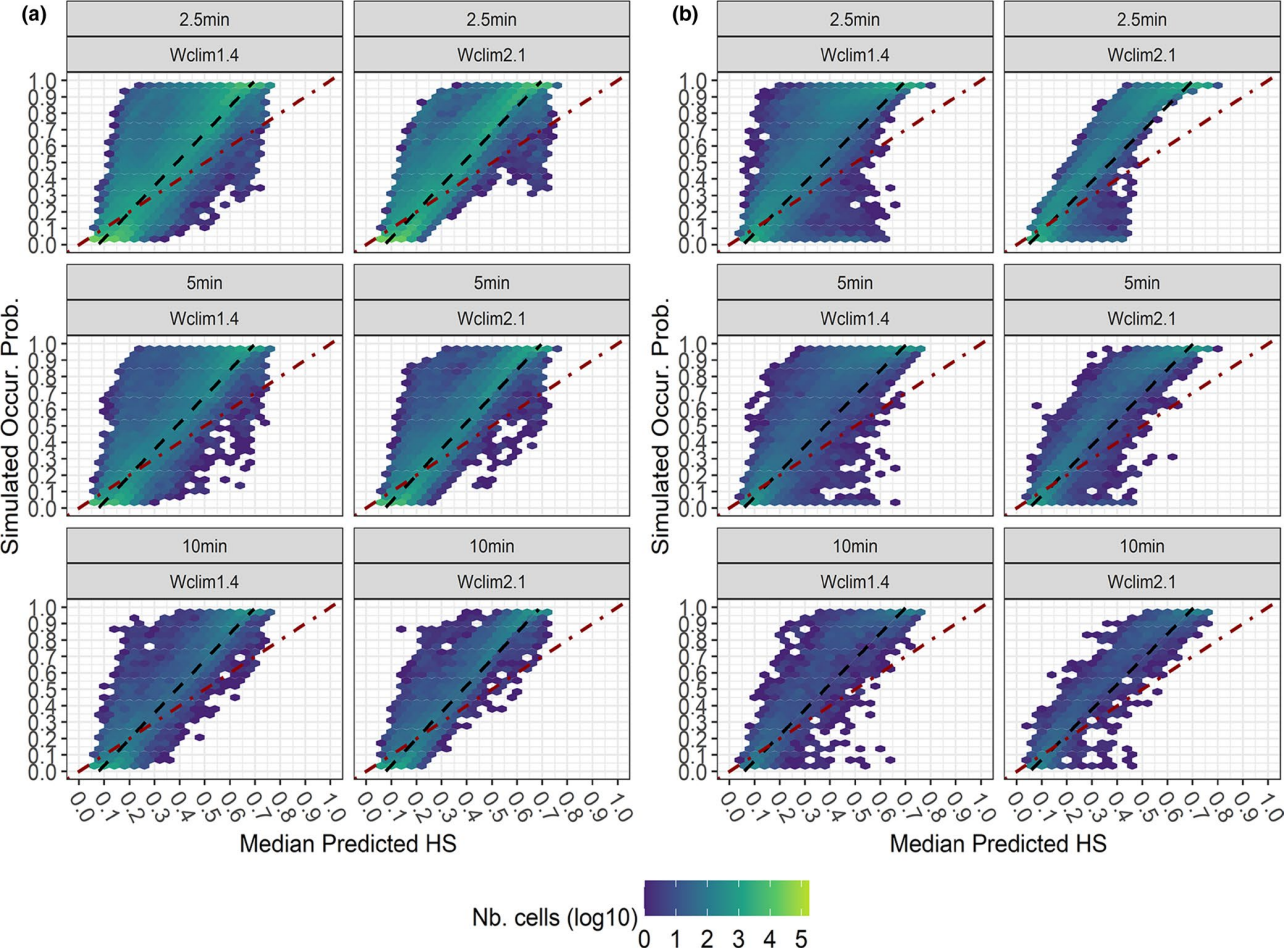
Hexagonal heatmaps (width of each hexagonal cell = 0.04) showing, for each Worldclim version * Grid resolution combination, the degree of correspondence between Simulated Occurrence Probability and Median predicted HS for the (a) Alpine and (b) Mediterranean VSs.; for each hexagonal cell, log10 of the number of pixels whose occurrence probability‐median HS pair of values fall within the cell itself is reported in a blue‐to‐yellow scale; the dot‐dashed diagonal red line shows theoretical 1:1 predicted‐simulated correspondence; the dashed black line represents smoothed regression line from the linear model Simulated Occurr. Prob. ~ Median predicted HS, summarizing the trend emerging from the heatmap

**FIGURE 7:**
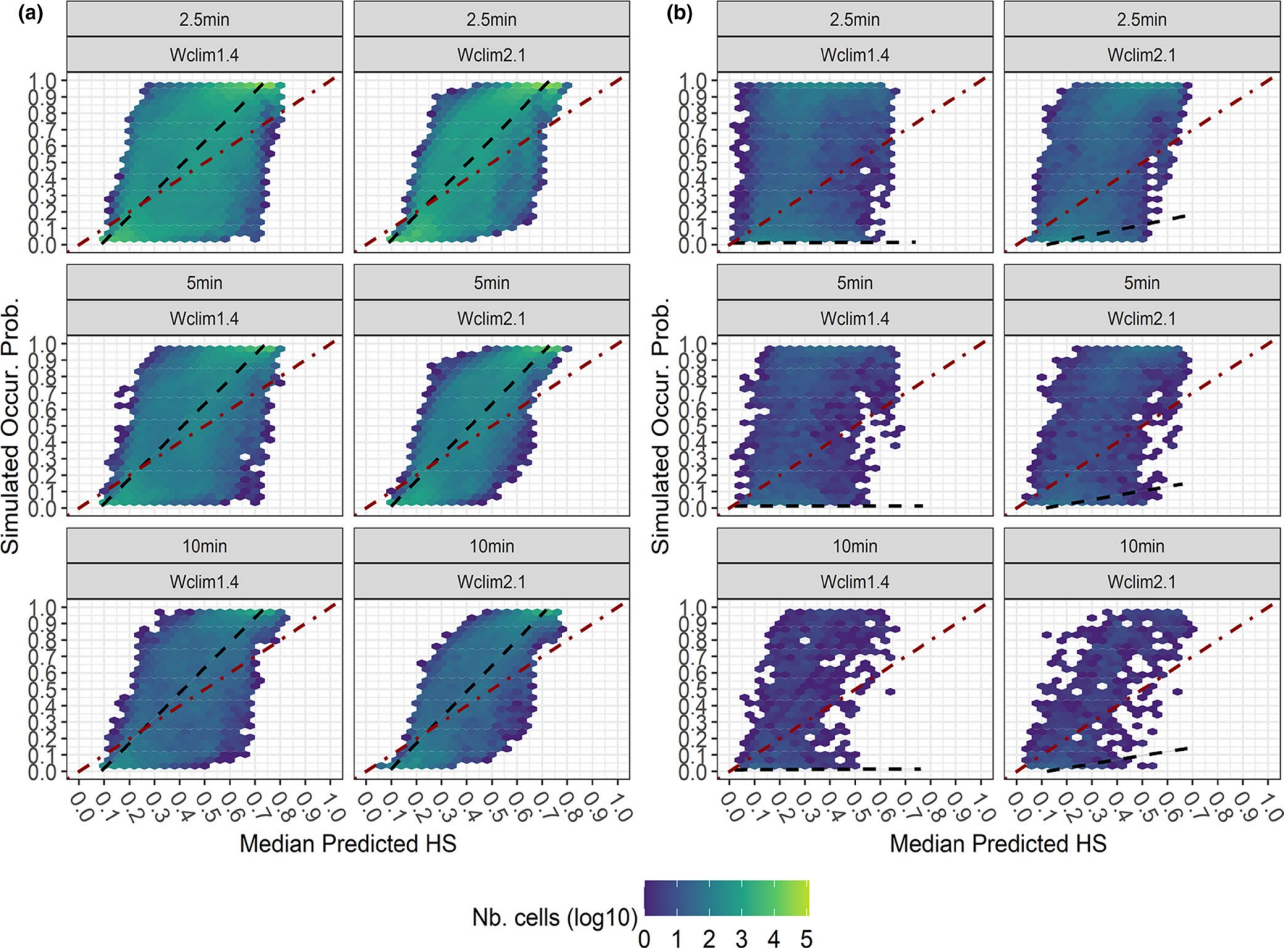
Hexagonal heatmaps (width of each hexagonal cell = 0.04) showing, for each Worldclim version * Grid resolution combination, the degree of correspondence between Simulated Occurrence Probability and Median predicted HS for the (a) Generalist and (b) Restricted VSs.; for each hexagonal cell, log10 of the number of pixels whose occurrence probability‐median HS pair of values fall within the cell itself is reported in a blue‐to‐yellow scale; the dot‐dashed diagonal red line shows theoretical 1:1 predicted‐simulated correspondence; the dashed black line represents smoothed regression line from the linear model Simulated Occurr. Prob. ~ Median predicted HS, summarizing the trend emerging from the heatmap

Although overall correlation between median predicted HS and simulated occurrence probability was high for all the VSs except the Restricted one (Appendix Table S2), median predicted HS did not tightly approach the 1:1 correspondence with simulated occurrence probability for any VS * Worldclim version * grid resolution combination (Figures 6 and 7). Nonetheless, the Alpine and Mediterranean VSs showed a closer predicted‐simulated correspondence than the Generalist and Restricted ones, as a noticeably higher number of pixels with high median HS (although HS_max_ = 0.7–0.8) corresponding to high simulated occurrence probability, as well as of pixels with low median HS corresponding to low occurrence probability, appeared for the formers. The Restricted VS showed very low predicted‐simulated correspondence for Wclim2.1‐based projections, and almost none for Wclim1.4‐based ones (Figure 7b). Wclim2.1‐based median projections showed lower overdispersion of pixels around the 1:1 line than Wclim1.4‐based ones at 2.5 min, particularly for the Mediterranean VS (Figure 6b), while this difference became milder at 5 min and 10 min.

**FIGURE 8:**
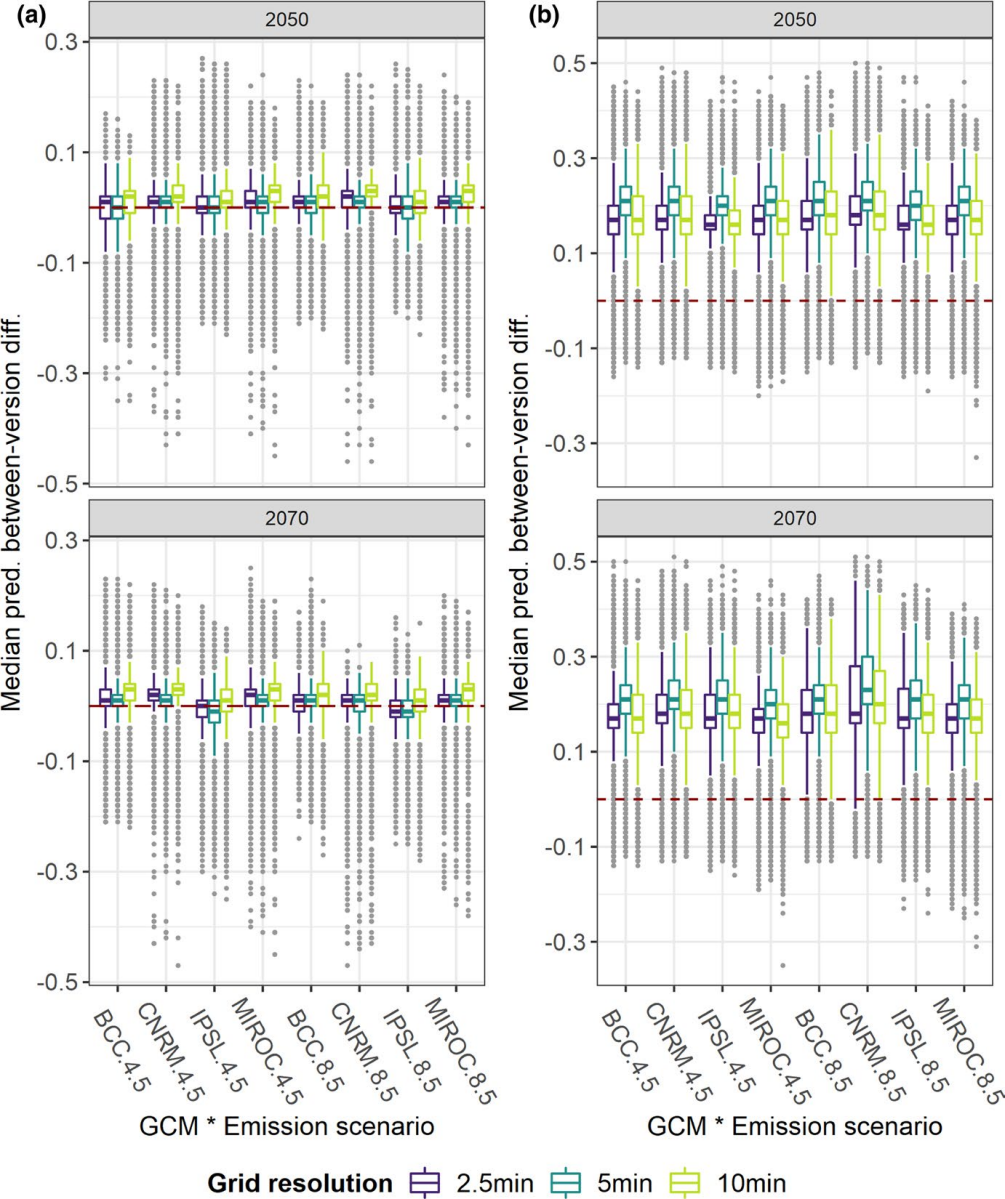
Boxplots showing between‐version difference (HSMs from Worldclim 2.1 ‐ HSMs from Worldclim 1.4) in median HS computed from HSMs' projections to the considered future climatic scenarios for the (a) Alpine and (b) Mediterranean VSs. Grey dots represent outliers

**FIGURE 9:**
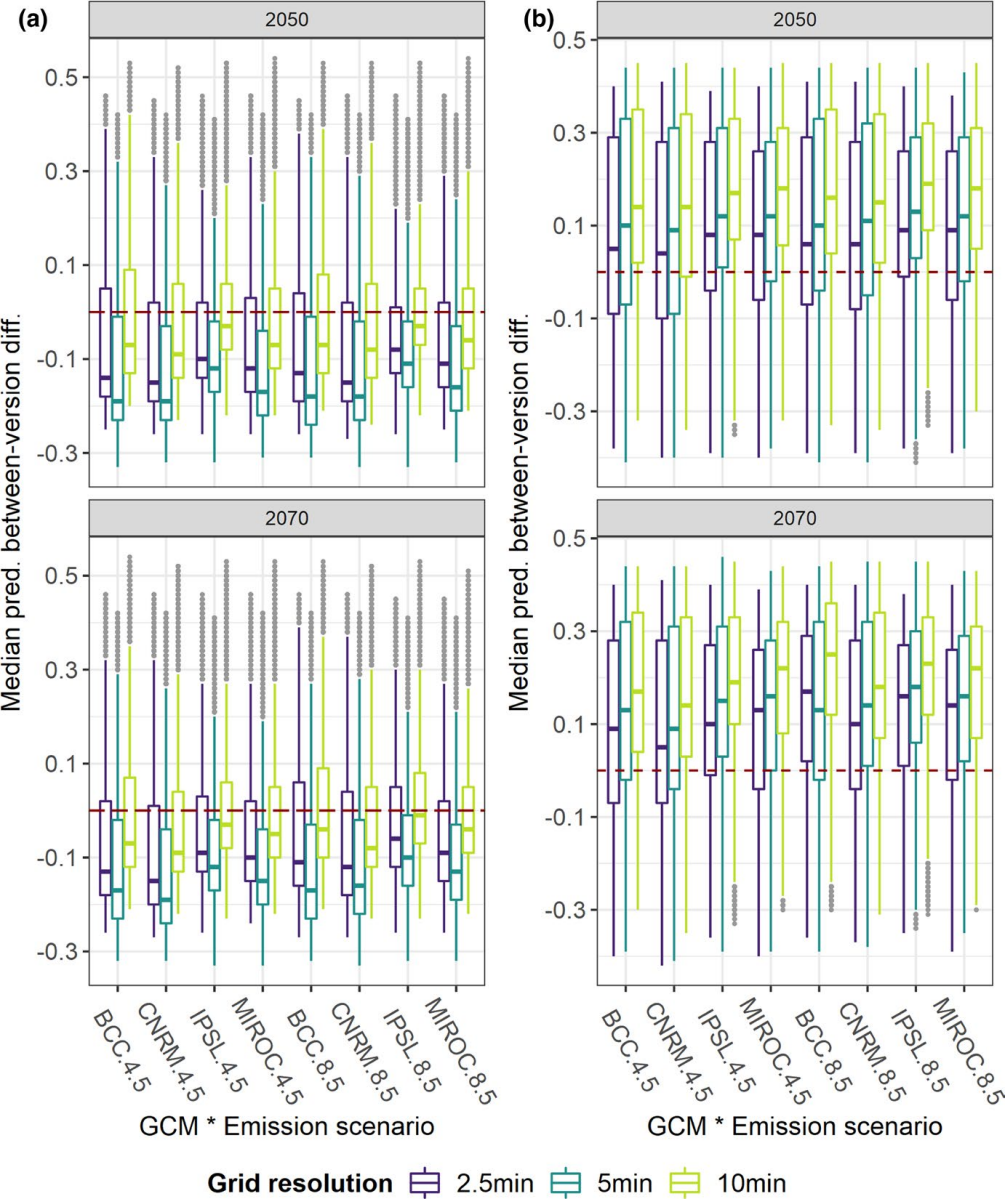
Boxplots showing between‐version difference (HSMs from Worldclim 2.1 ‐ HSMs from Worldclim 1.4) in median HS computed from HSMs' projections to the different considered future climatic scenarios for: (a) Generalist VS; (b) Restricted VS. Grey dots represent outliers

Between‐version differences in median predicted HS under the considered future scenarios were negligible for the Alpine VS regardless of grid resolution (Figure 8a), while Wclim2.1‐based median HS exceeded Wclim1.4‐based one (median difference = 0.15–0.2) for the Mediterranean VS across all the timeframe * GCM * emission scenario * grid resolution combinations (Figure 8b). Wclim2.1‐based median projections mainly underpredicted HS compared to Wclim1.4‐based ones under all future scenarios for the Generalist VS (Figure 9a), while the opposite emerged for the Restricted one (Figure 9b), although variability in between‐version difference values was noticeably higher for these VSs than for the Alpine and Mediterranean ones. Some discrepancies among the selected GCMs emerged for the Alpine and Generalist VSs, with IPSL resulting in between‐version differences somewhat diverging from the other GCMs. Moreover, in 2070, between‐version differences for the Mediterranean and Restricted VSs were higher under Scen8.5 than under Scen4.5 within most GCM * grid resolution combinations. Focusing on grid resolution, between‐version differences under future climate were higher for 5 min than for 2.5 min and 10 min when looking at the Mediterranean and Generalist VSs, while higher differences emerged for 10 min considering the Restricted VS.

### 3.5 Importance of factors

**FIGURE 10:**
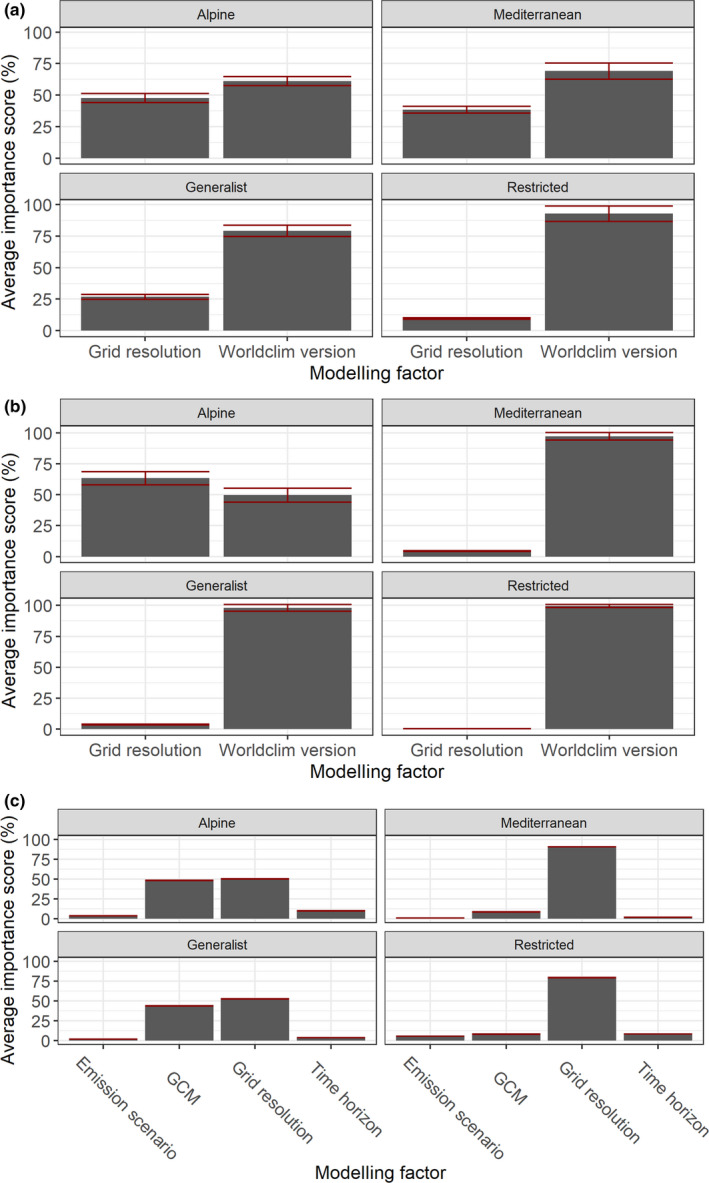
Average (± standard deviation, red bars) importance, for each VS, of the considered factors upon modelling outcomes, computed through the permutation‐based (*n* = 5) procedure implemented in the ‘biomod2’ R package and applied to Random Forests regression models fitted with response variable set to: (a) standardized importance scores of input variables within the HSMs exceeding the chosen AUC and TSS thresholds; (b) correlation between median predicted HS and the simulated occurrence probability of the considered VS; (c) between‐version differences in median predicted HS under the considered future climatic scenarios

When included as factor in RF models fitted with either predicted‐simulated *r* or future between‐version differences as response variable, the considered VS attained by far the highest average importance score (94.7% ± 3.7% for predicted‐simulated *r*; 96.5% ± 0.2% for between‐version differences in future projections). When fitting RF models for each VS separately, average importance score of Worldclim version was clearly higher than that of grid resolution, considering Std_Imp (Figure 10a) or predicted‐simulated *r* (Figure 10b) as response variable, for all the VSs except the Alpine one; for this latter, grid resolution appeared more important than Worldclim version for predicted‐simulated *r*, while the opposite resulted for Std_Imp. Finally, grid resolution emerged for all the VSs as the preponderant factor influencing between‐version differences in median HS under future climate, closely followed by GCM for the Alpine and Generalist VSs (Figure 10c).

## 4 DISCUSSION

The pervasive use of Worldclim during the last fifteen years to estimate climate‐occurrence relationships for a huge variety of taxa calls for the need to assess magnitude, location, and drivers of prediction discrepancies between models fitted using climate surfaces derived from the first version of this database (Worldclim 1.4) and those relying upon the recently updated one (Worldclim 2.1). For instance, in recent years geographical predictions from HSMs implementing Wclim1.4‐derived climate surfaces have frequently guided, at least partially, the identification of areas deserving attention as potentially suitable for rare and endangered species (Console et al., 2020; Forrest et al., 2012; Iannella et al., 2018; Marini et al., 2009) or, on the other hand, for invasive ones (Cerasoli et al., 2019; Di Febbraro et al., 2019; Ficetola et al., 2009). In case prioritization measures are conceived based on these predictions, *a priori* knowledge about which areas will show the greatest changes in predicted suitability in case the updated Worldclim variables are used would be useful to refine such measures. Indeed, although climate databases based on quasi‐mechanistic downscaling such as CHELSA (Karger et al., 2017) or remote sensing like MERRACLIM (G. C. Vega et al., 2017) may be more accurate than Worldclim in regions with scarce meteorological stations (e.g. polar regions or tropical rainforests) (Morales‐Barbero & Vega‐Álvarez, 2019), Worldclim interpolated climate surfaces represent an invaluable data source in station‐richer regions as North America, south‐eastern Africa and most of the Palearctic (Fick & Hijmans, 2017). Here, we investigated how niche characteristics of the target species, grid resolution and projection scenarios influence between‐version differences in Worldclim‐based HSMs.

Limiting the “virtual sampling” to a buffer around occurrence areas for the three climate‐constrained VSs permitted to obtain sample prevalences matching the “safety range” (i.e. 0.1–0.9) recommended by Jiménez‐Valverde (2021) to get unbiased estimations of model discrimination capacity with presence‐absence data. Thus, we can be confident that the HSMs selected for the model projection phase, and from which between‐version differences in predicted HS were assessed under current and future time horizons, were actually the ones attaining a good predictive performance.

Contrasting the Generalist VS with the others, considerably fewer HSMs attained sufficient discrimination performance to enter the projection phase; this, coupled with higher RMSE, further corroborates mounting evidence about the difficulties in modeling the ecological requirements of euryoecious species (Cerasoli et al., 2017; Connor et al., 2018; Santini et al., 2021). On the other hand, the degree to which our HSMs properly estimated the simulated climate‐occurrence relationships varied among the three climate‐constrained VSs: indeed, the HSMs fitted for the Alpine and Mediterranean VSs succeeded in isolating the variables influencing their suitability while the models obtained for the Restricted one underestimated the influence of the variables (especially bio2) more directly favoring its occurrence. Such difference translated into the relatively high correspondence between median predicted suitability and simulated occurrence probability emerging for the Alpine and Mediterranean VSs and into the almost absent correspondence characterizing the Restricted one. These patterns may be because suitability for the Restricted VS was simulated as dependent upon a combination of climatic conditions very poorly represented in Europe, resulting in the lowest prevalence among all the VSs, both across Europe and within Pres‐Abs samples; this in turn forced the corresponding HSMs to estimate its climate‐occurrence relationships with few presences available and, arguably, to widely extrapolate when projected.

Generally, median predicted suitability more closely approached simulated occurrence probability when Wclim2.1‐derived variables were used. This was somehow expected, as occurrence probability for our VSs was simulated using Wclim2.1‐derived climate surfaces, at 2.5 min resolution, as reference layers. However, this between‐version difference was not equally apparent for all the VSs: while it was substantial for the Mediterranean VS, in the case of the Alpine one grid resolution contributed more than Worldclim version in determining closeness of median predictions to simulated occurrence probability. Interestingly, the predicted‐simulated correspondence did not worsen for any VS at increasing grain size (i.e., at 5 min and 10 min). This result partially contrasts with previous studies suggesting a deterioration of HSMs' predictions when fitted at coarser spatial resolution than that at which real‐world species react to environmental changes (Seo et al., 2009; Vale et al., 2014) or at which VSs' suitability patterns are simulated (Connor et al., 2018), although these works also highlighted that such deterioration greatly vary across species and ratios of coarser‐to‐correct grid resolutions.

Between‐version local correlation in values of the selected bioclimatic variables was relatively high across most of Europe considering yearly (i.e., bio1) and seasonal (i.e., bio8, bio18, and bio19) temperature and precipitation averages, while climate surfaces from the two Worldclim versions showed a number of lowly correlated areas for variables representing diurnal (i.e., bio2) or yearly (i.e., bio4 and bio15) variability, particularly at 2.5 arc‐minutes. This likely explains why model projections resulted in a major portion of Europe showing diverging suitability for the Restricted (primarily responding to bio2) and Generalist (mainly associated to bio4) VSs, while between‐version discrepancies for the Alpine and Mediterranean VSs mainly involved scattered regions characterized by high occurrence probability for these latter and corresponding to relatively extreme average temperature/precipitation values. Thus, our results show that, at the European scale, HSMs fitted using Wclim2.1‐derived climate surfaces may produce considerably divergent spatial predictions compared to models fitted using Worldclim 1.4 for species whose climate‐occurrence relationships are driven by variability in temperature/precipitation regimes. Differently, for species mainly responding to yearly or seasonal average temperature/precipitation trends attention should be primarily paid to areas showing extreme values (e.g. mountainous massifs for bio1, southern Europe for bio18).

Looking at future scenarios, differences in climate‐occurrence relationships guided between‐version mismatch in predicted suitability: predictions derived from the two Worldclim versions appear convergent for species inhabiting mountainous areas and high latitudes (Alpine VS), Wclim2.1‐derived HSMs mainly underpredict suitability compared to Wclim1.4‐derived ones for euryoecious species (Generalist VS), while the opposite emerge for species inhabiting southern Europe (Mediterranean and Restricted VSs). Interestingly, HSMs fitted at 5 min for the Mediterranean and Generalist VSs led to higher between‐version differences in future suitability compared to models fitted at 2.5 min and 10 min, while higher differences resulted at 10 min for the Restricted VS. Since across‐resolution differences did not clearly emerge under present conditions, these results suggest that possible changes in correlation among predictors under future scenarios (Mesgaran et al., 2014; Zurell et al., 2012) may interact with grid resolution in influencing prediction discrepancies between models derived from the two Worldclim versions. Moreover, the overall change in the sign of between‐version differences for the Restricted VS contrasting the current scenario (Wclim1.4‐derived HS higher than Wclim2.1‐derived one across wide extents of eastern Europe) with the future ones (Wclim1.4‐derived HS lower than Wclim2.1‐derived one within most of the pixels sampled across Europe) suggests that discrepant changes between the two Worldclim versions in the values of some climate surfaces from current to future scenarios, combined with the presumably extensive extrapolation that the HSMs faced due to the limited calibration area, made predictions for this VS particularly sensitive to the chosen climate data source. Translated into real‐world situations, this result should further warn about the need to attentively analyze predictions of future potential distributional shifts from climate‐based HSMs fitted for currently narrow‐ranging species (Santini et al., 2021). Quite reassuringly, neither the specific emission scenario nor the chosen GCMs dramatically influenced between‐version differences in future projections.

In conclusion, implementing the virtual ecologist approach, we highlighted that, depending primarily on the species' climatic tolerances and secondly on the grain size of the climate surfaces, prediction mismatches between HSMs fitted on bioclimatic variables from the two Worldclim versions considerably vary across Europe, providing useful information to re‐evaluate results of previous works based on Worldclim data. We invite researchers to replicate and possibly improve, for instance including also instances of species‐climate disequilibrium, our simulations within other continents to assess if our findings change under different baseline climate gradients and density of meteorological stations.

## CONFLICT OF INTEREST

The Authors declare no conflict of interest.

## AUTHOR CONTRIBUTIONS

**Francesco Cerasoli:** Conceptualization (equal); data curation (lead); formal analysis (lead); investigation (equal); methodology (lead); writing – original draft (equal); writing – review & editing (equal). **Paola D'Alessandro:** Conceptualization (equal); data curation (supporting); formal analysis (supporting); investigation (equal); methodology (supporting); writing – original draft (equal); writing – review & editing (equal). **Maurizio Biondi:** Conceptualization (equal); data curation (supporting); formal analysis (supporting); investigation (equal); methodology (supporting); writing – original draft (equal); writing – review & editing (equal).

## Supporting information

Supplementary Material

## Data Availability

Coordinates (EPSG3035: ETRS89 – LAEA) of the presence‐absence records generated for each virtual species * sampling replicate combination, along with the raster files representing simulated occurrence probability and presence‐absence of the virtual species across Europe, and the R script written to conduct all the analyzes reported in the present article, are freely downloadable from the Dryad online repository (https://doi.org/10.5061/dryad.8w9ghx3nr).
