# Supplementary material for: Worldclim 2.1 versus Worldclim 1.4: Climatic niche and grid resolution affect between‐version mismatches in Habitat Suitability Models predictions across Europe
Source: Ecol Evol. 2022 Feb 14;12(2):e8430. doi: 10.1002/ece3.8430 (PMC8844118; doi:10.1002/ece3.8430)
Supplement: Supplementary file 1 — Supplementary Material [file ECE3-12-e8430-s001.docx]

**Appendix**

**Note S1.** Parameterizations of the customized response functions and PCA used to simulate Habitat Suitability (HS) for the four virtual species (VS) through the ‘virtualspecies’ R package (version 1.5.1).

- Alpine VS:

‘generateSpFromFun’ (bio1: ‘logisticFun’, alpha = 2.5, beta = 3; bio8: ‘quadraticFun’, a = -0.004, b = 0.05, c = 0.7; bio2: ‘logisticFun’, alpha = -1.5, beta = 10; formula = 2.5*bio1+1.5*bio8+0.5*bio2).

- Mediterranean VS:

‘generateSpFromFun’ (bio1: ‘logisticFun’, alpha = -2, beta =12.5; bio18: ‘logisticFun’, alpha = 2.5, beta = 110; formula = bio1+0.75*bio18).

- Generalist VS:

‘generateSpFromPCA’ (niche.breadth=’wide’, sample.points = T, nb.points = 50000).

- Restricted VS:

‘generateSpFromPCA’ (niche.breadth=’narrow’, sample.points = T, nb.points = 100000).

**Note S2.** Model parameterization of: ‘full-data’ Habitat Suitability Models (HSMs) fitted on the 50 complete presence-absence (Pres-Abs) datasets generated for each VS; biomod2-based HSMs fitted, for each of the 50 Pres-Abs datasets, upon training data selected through checkerboard spatial blocking.

- ‘full-data’ HSMs:

1. GAM: ‘gam’ R package, function ‘step.Gam’ (family = ‘binomial’, formula: PresAbs ~ bio1+bio2+bio4+bio8+bio15+bio18+bio19, arg = c(‘df’ = 2, ‘df’ = 3), direction = ‘both’).
2. GBM: Elith et al. (2008), function ‘gbm.step’ (gbm.y = PresAbs; gbm.x = ModellingVars; tree.complexity=3, learning.rate=0.001, max.trees=10000, n.folds=10), with ‘ModellingVars’ being bio1, bio2, bio4, bio8, bio15, bio18 and bio19.

Elith, J., Leathwick, J. R., & Hastie, T. (2008). A working guide to boosted regression trees. *Journal of animal ecology, 77*(4), 802-813.

- biomod2-based HSMs:

‘biomod2’ R package, function ‘BIOMOD_ModelingOptions’ (GAM = list(algo=GAM_mgcv, interaction.level=2), GLM=list(type='quadratic', interaction.level=2), GBM=list(n.trees=10000, interaction.depth=3, cv.folds=10, bag.fraction=0.75), RF=list(ntree=5000))

**Fig. S1.** Local correlation coefficient (Pearson’s *r*) across Europe between values from Worldclim 1.4 and those from Worldclim 2.1 of the seven selected bioclimatic variables at: **(a)** 2.5 arc-minutes grid resolution; **(b)** 5 arc-minutes resolution; **(c)** 10 arc-minutes resolution. Local correlation was computed using the ‘corLocal’ function from the ‘raster’ R package (version 3.3-13), with neighbourhood size set to 3*3 pixels (i.e. ‘ngb’ = 3). Before computing local correlation, raster maps from Worldclim 1.4 were resampled through bilinear interpolation to match dimensions of those from Worldclim 2.1.


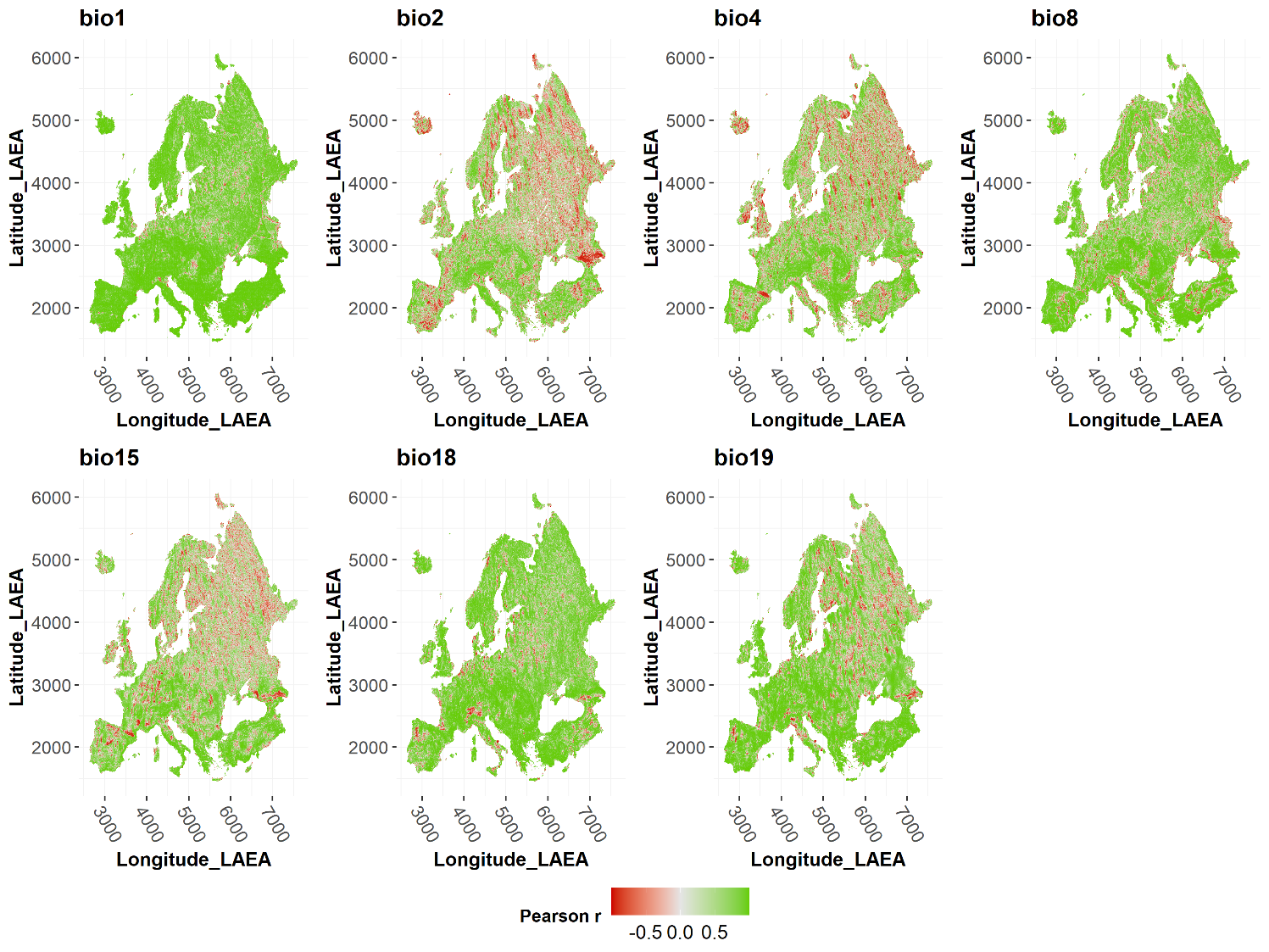
**(a)**

**Fig. S1.** (continues from previous page)

**
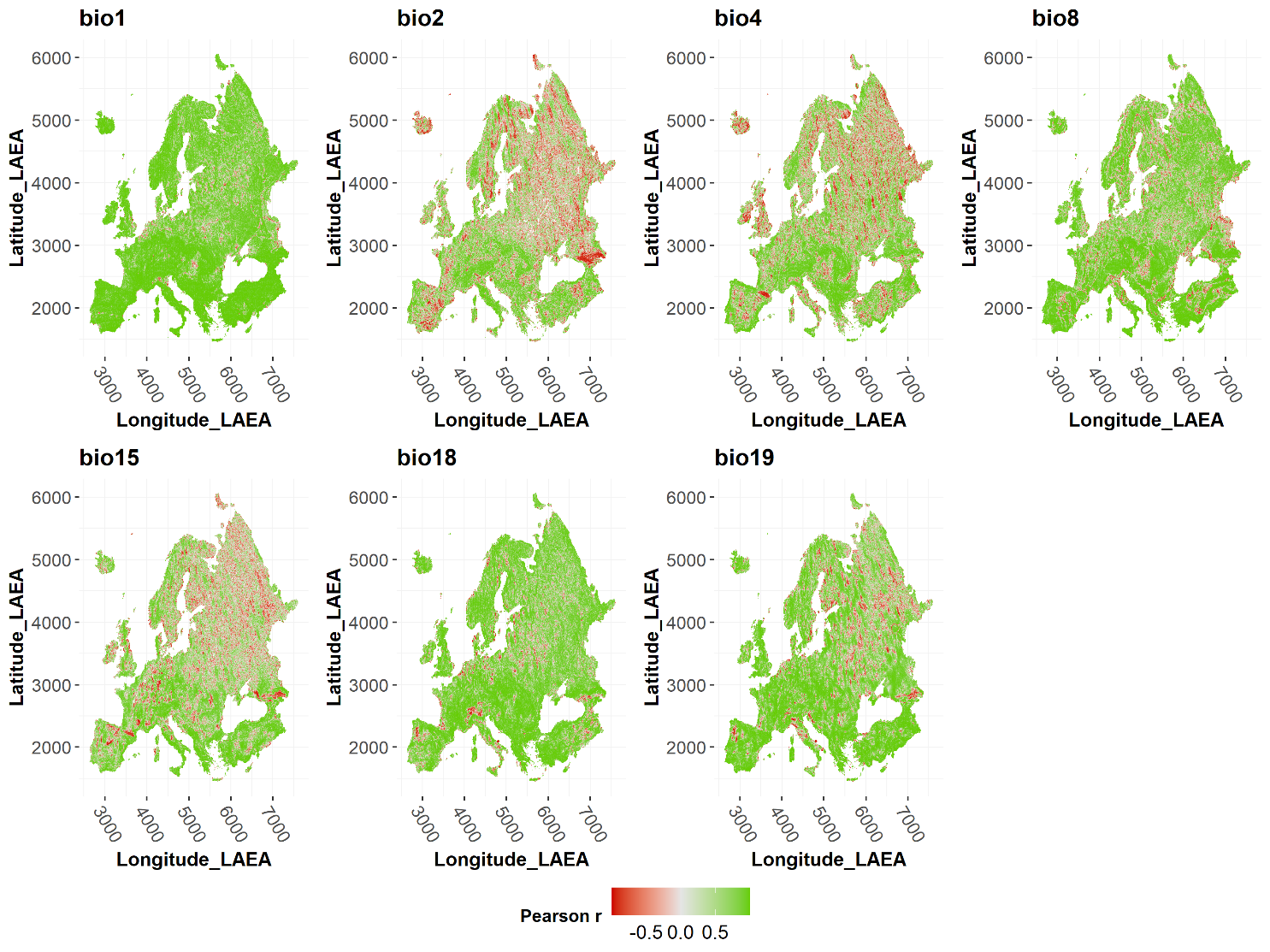
(b)**

**Fig. S1.** (continues from previous page)


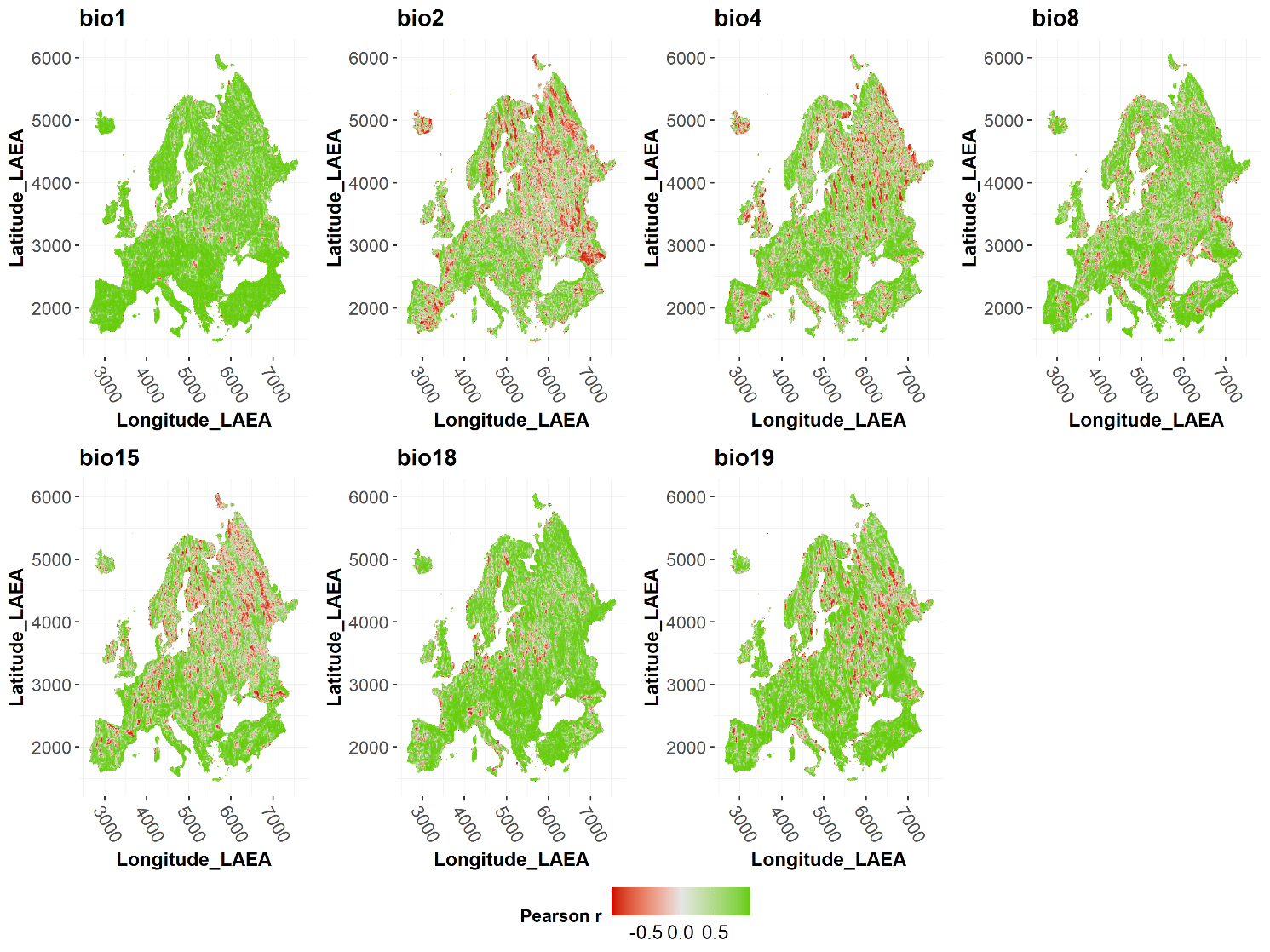
**(c)**

**Fig. S2.** Boxplots showing pairwise Pearson’s correlation coefficient (*r*) between the two Worldclim versions within 50 sets of points (*n* = 50000 for each set) sampled across Europe through regular random sampling (‘sampleRegular’ function from the raster R package version 3.3-13) for each variable * grid resolution combination. In (a) variable values were sampled from the ‘original’ layers of both Worldclim versions; in (b) the resampled Worldclim 1.4 layers were compared to the original ones
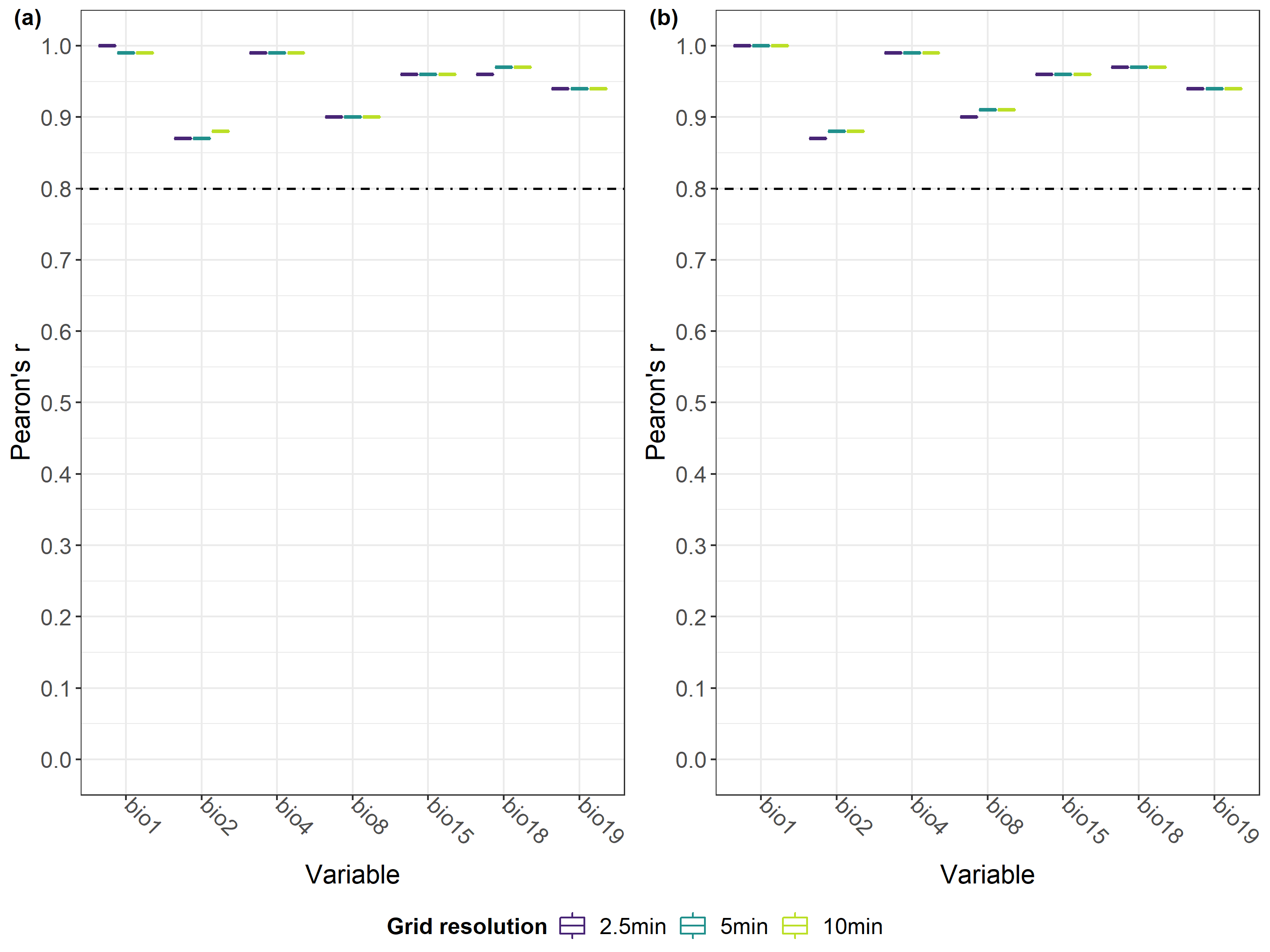
from Worldclim 2.1. Dash-dotted horizontal line indicates *r* = 0.8.


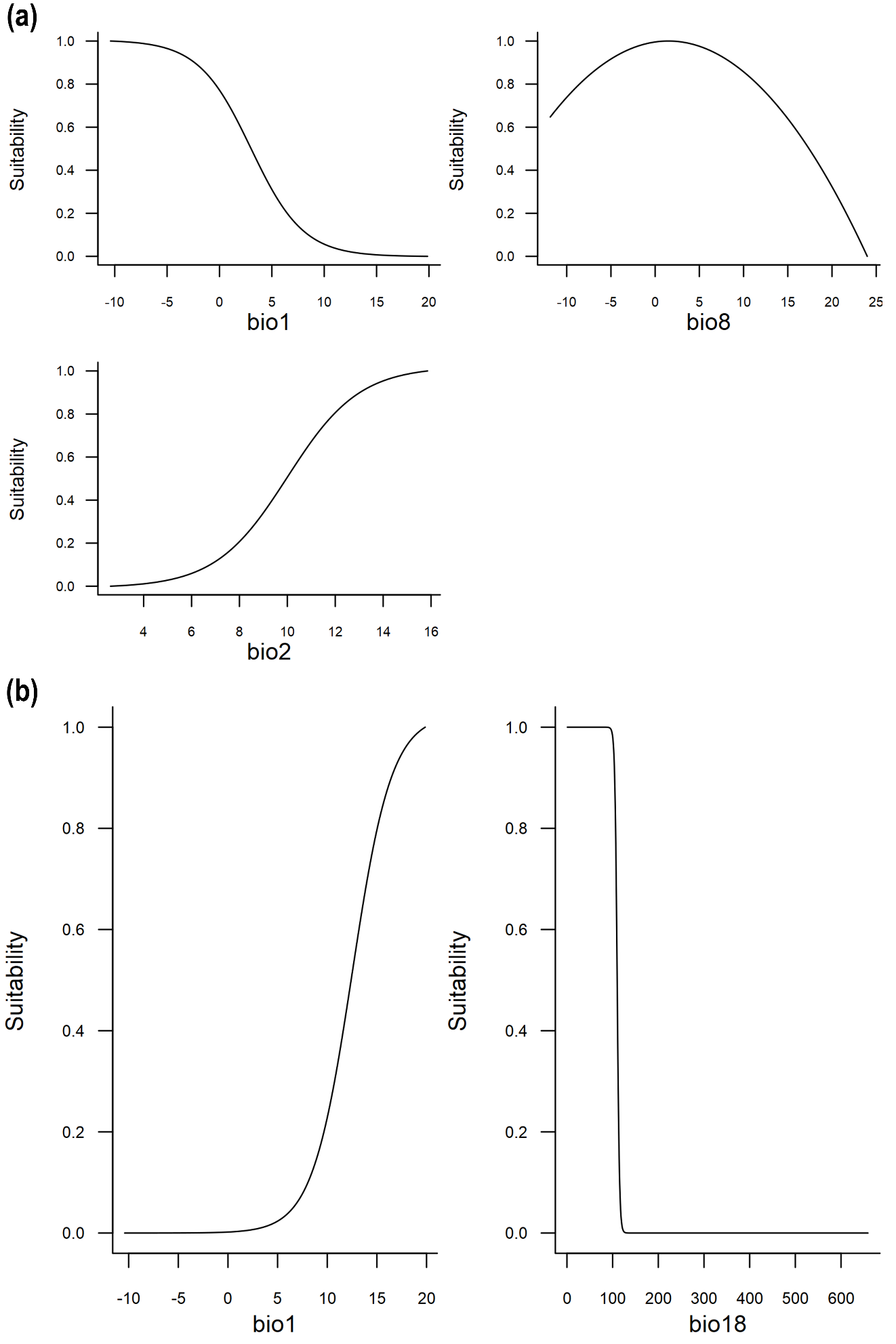
**Fig. S3.** Customized response curves used to simulate HS for: **(a)** Alpine VS and **(b)** Mediterranean VS.


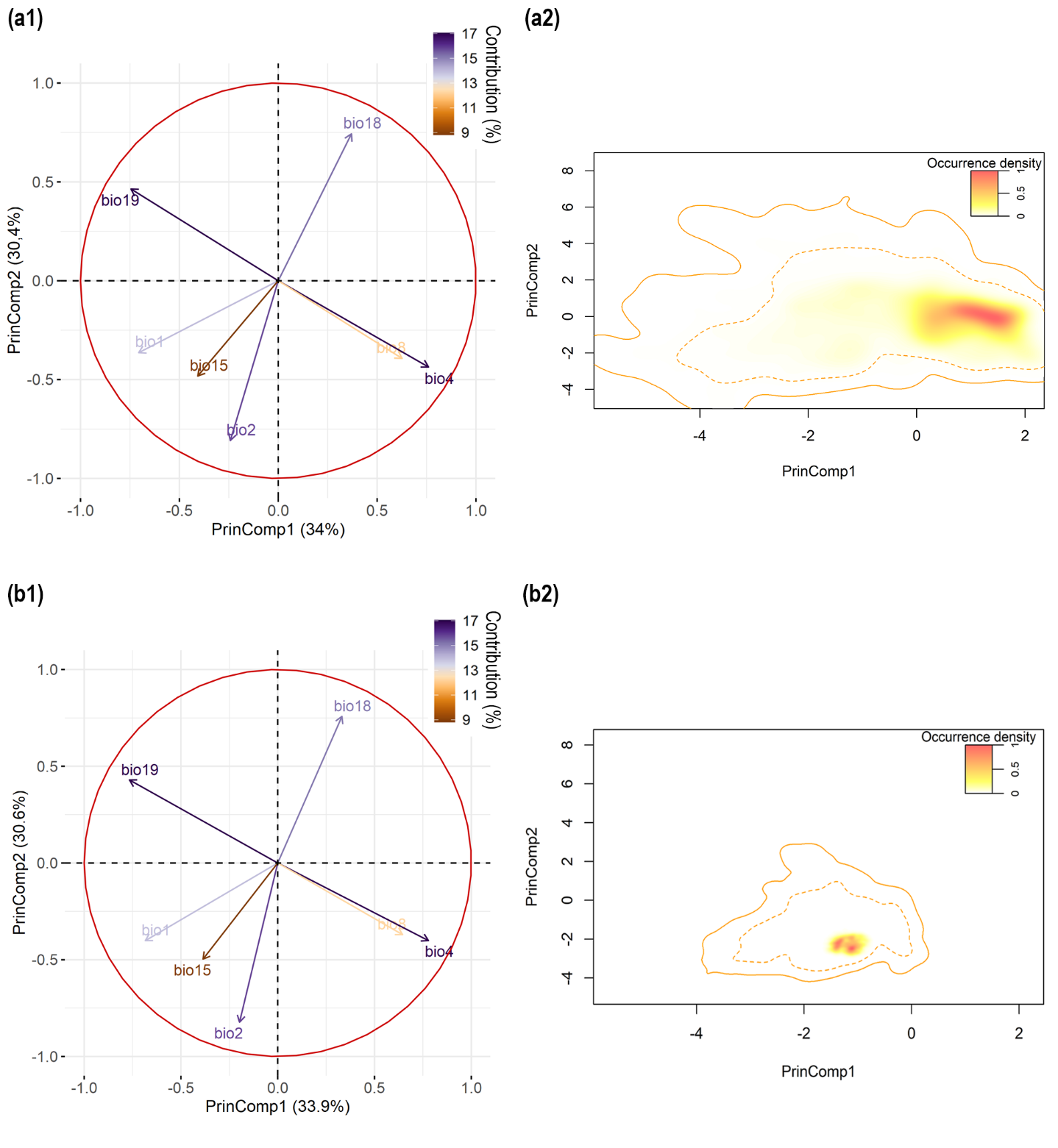
**Fig. S4.** Relative contribution of the selected input variables and density of occurrence of the VS within the PCA-derived 2D climatic space for: **(a1,2)** Generalist VS; **(b1,2)** Restricted VS.


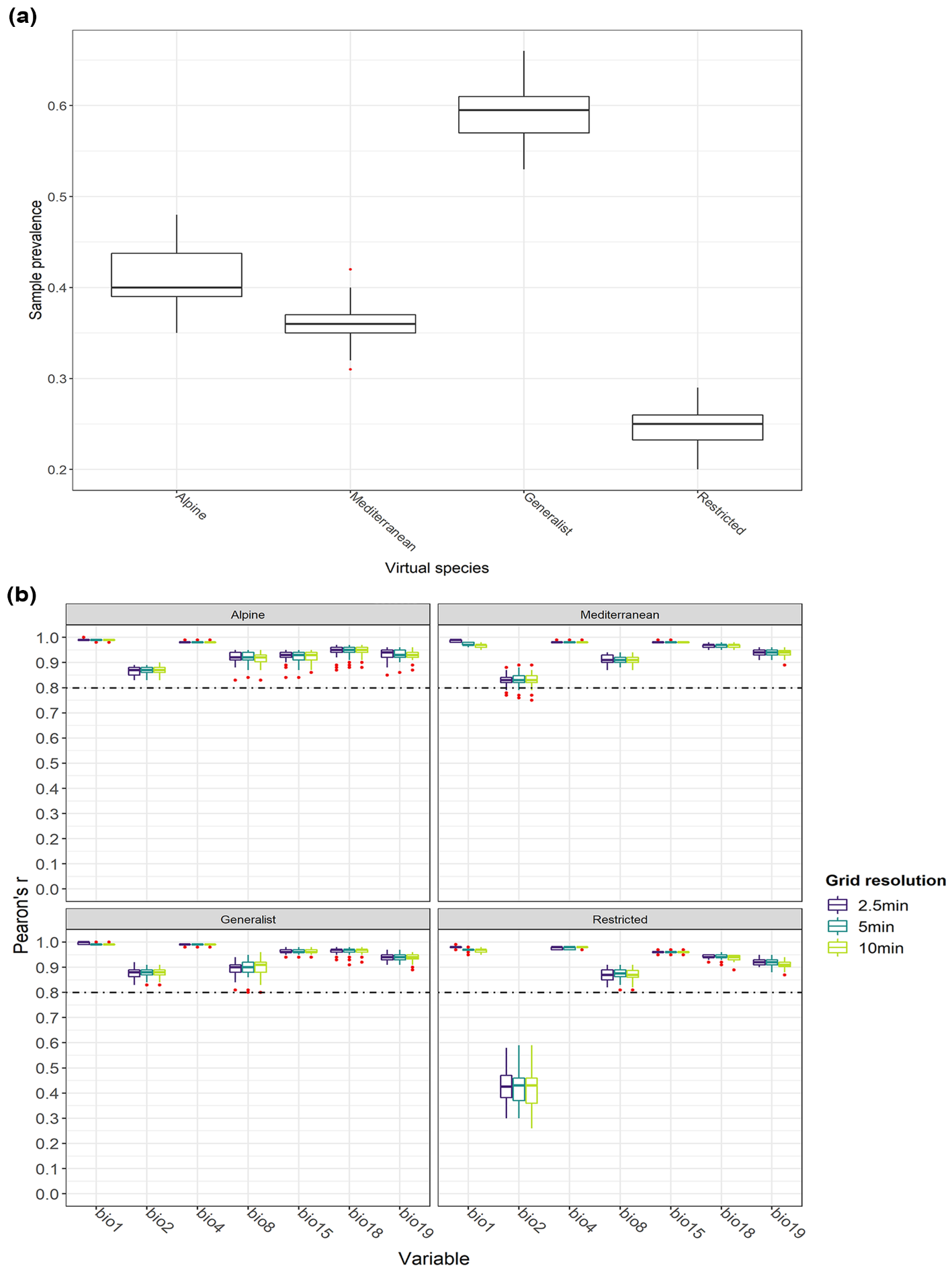
**Fig. S5. (a)** Boxplots showing, for each VS, sample prevalence across the 50 sampling replicates; **(b)** Pearson’s correlation coefficient between values from Worldclim 1.4 (resampled layers) and those from Worldclim 2.1 of the seven retained bioclimatic variables, sampled upon the presence-absence points, for each VS * grid resolution combination; dash-dotted horizontal line indicates *r* = 0.8.

**Fig. S6.** Correlograms showing spatial variation in Moran’s index (*I*) computed upon residuals from the ‘full-data’ HSMs fitted, for each Worldclim version * Grid resolution combination, for the **(a)** Alpine, **(b)** Mediterranean, **(c)** Generalist, and **(d)** Restricted VSs. Correlograms were obtained through the ‘correlog’ function from the ‘ncf’ R package (version 1.2-9), with the ‘increment’ parameter, representing the pace of incremental inter-point distance classes considered for Moran’s *I* computation, set to: 5 km when grid resolution was 2.5 arc-minutes, 10 km at 5 arc-minutes and 20 km at 10 arc-minutes. Dash-dotted red lines encompass Moran’s *I* = 0 ± 0.1.


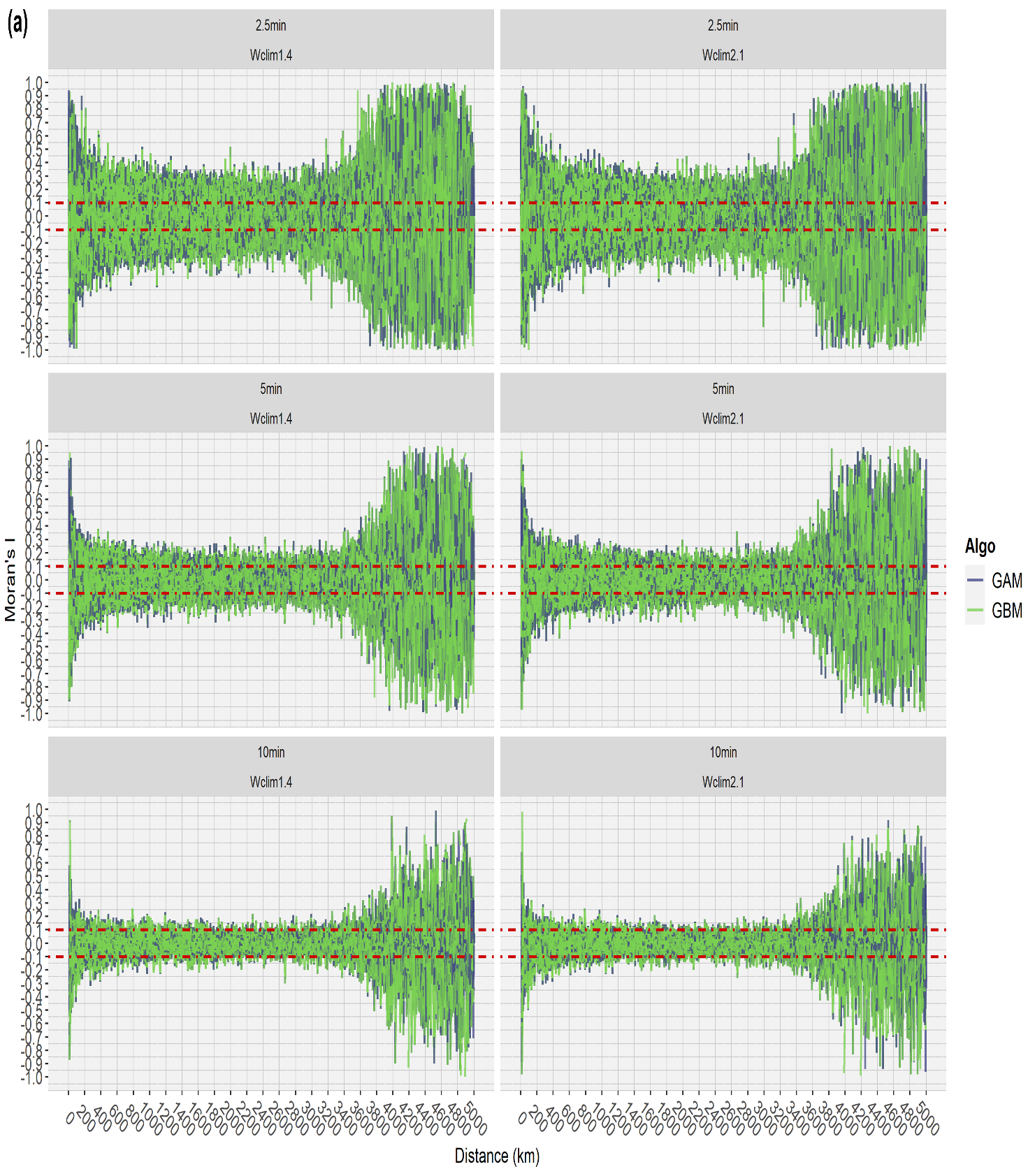


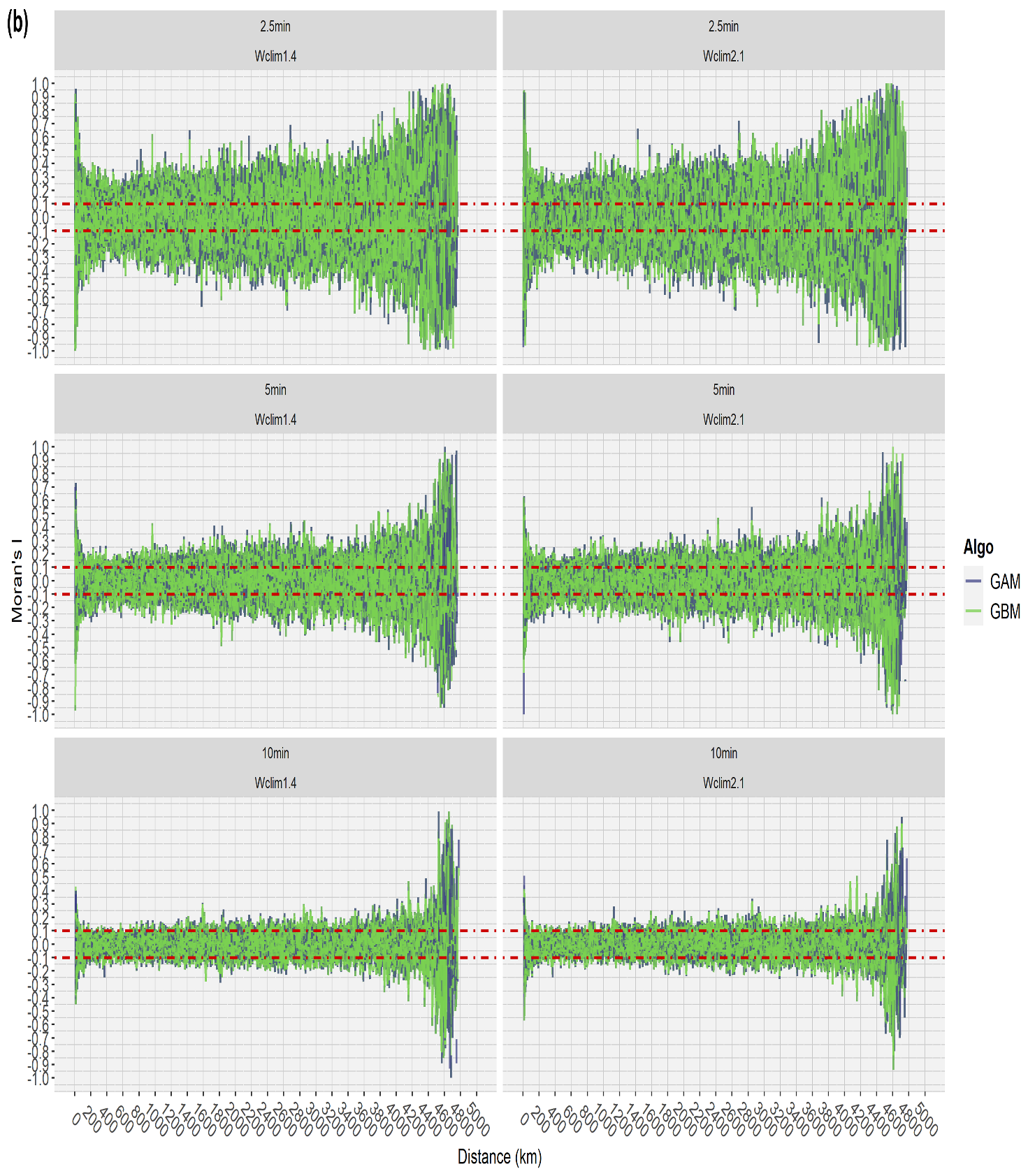
**Fig. S6.** (continues from previous page)


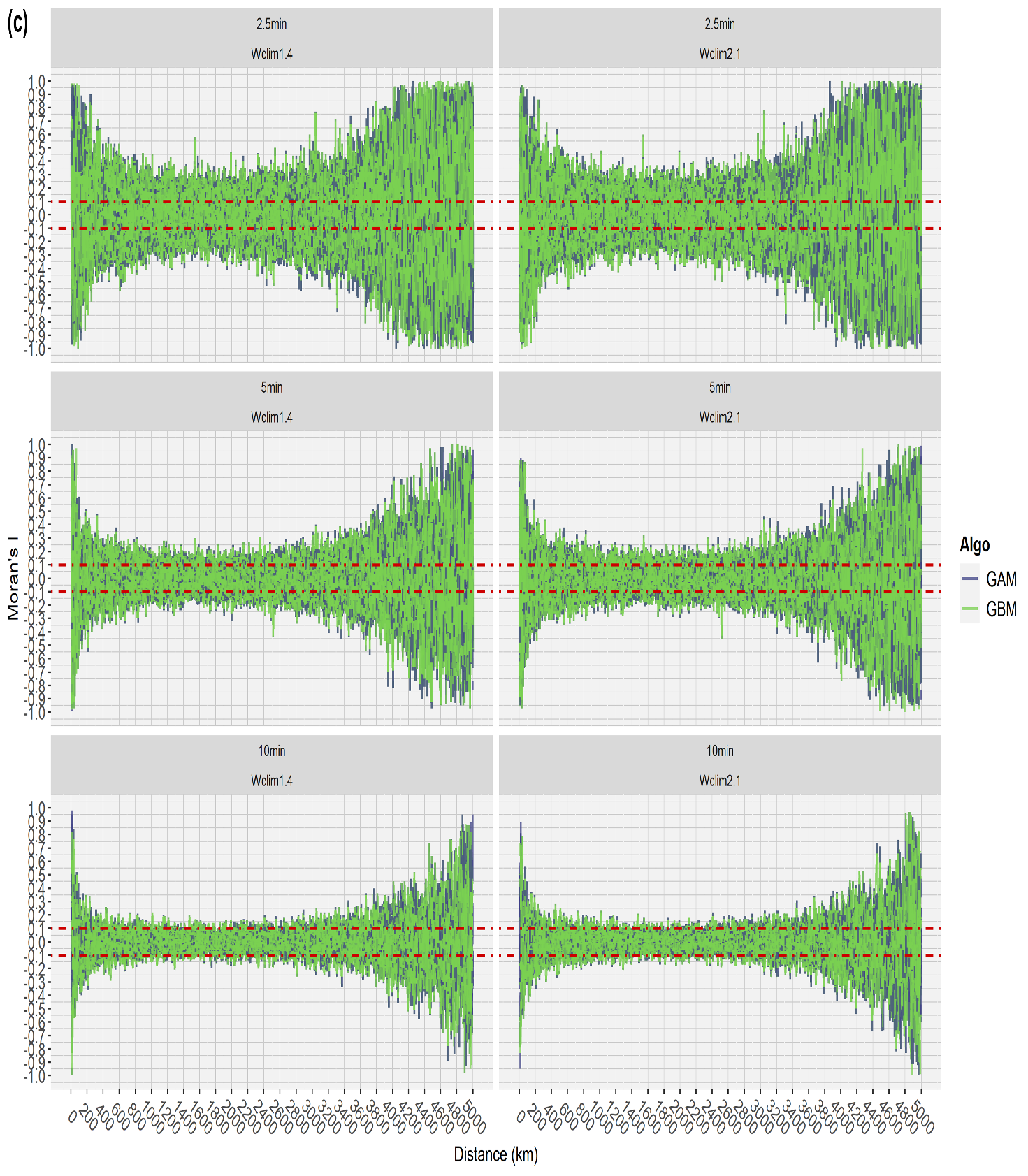
**Fig. S6.** (continues from previous page)


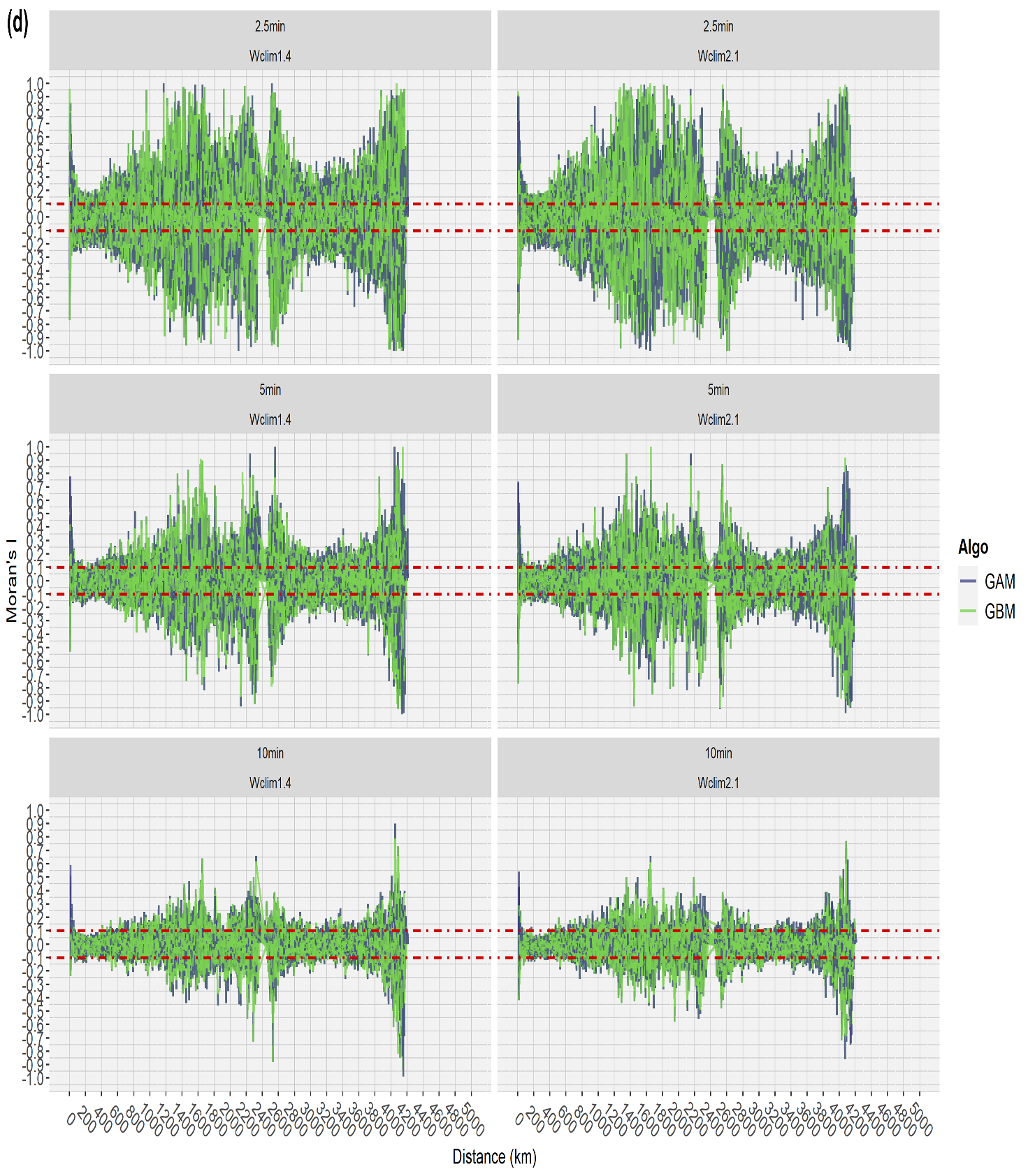
**Fig. S6.** (continues from previous page)


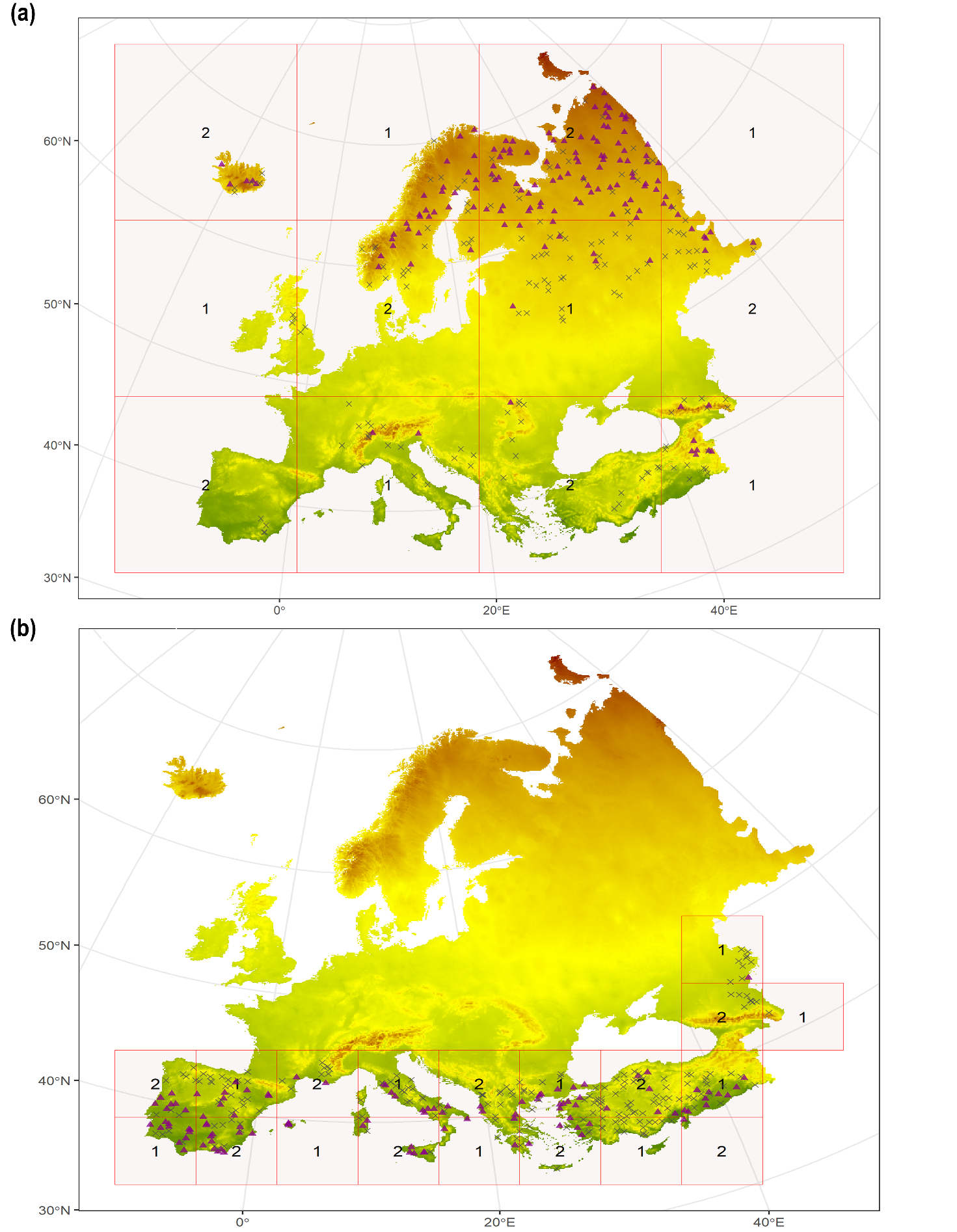
**Fig. S7.** Example of checkerboard spatial blocking obtained from one (randomly selected) of the 50 Pres-Abs samples for the **(a)** Alpine, **(b)** Mediterranean, **(c)** Generalist, and **(d)** Restricted VSs.


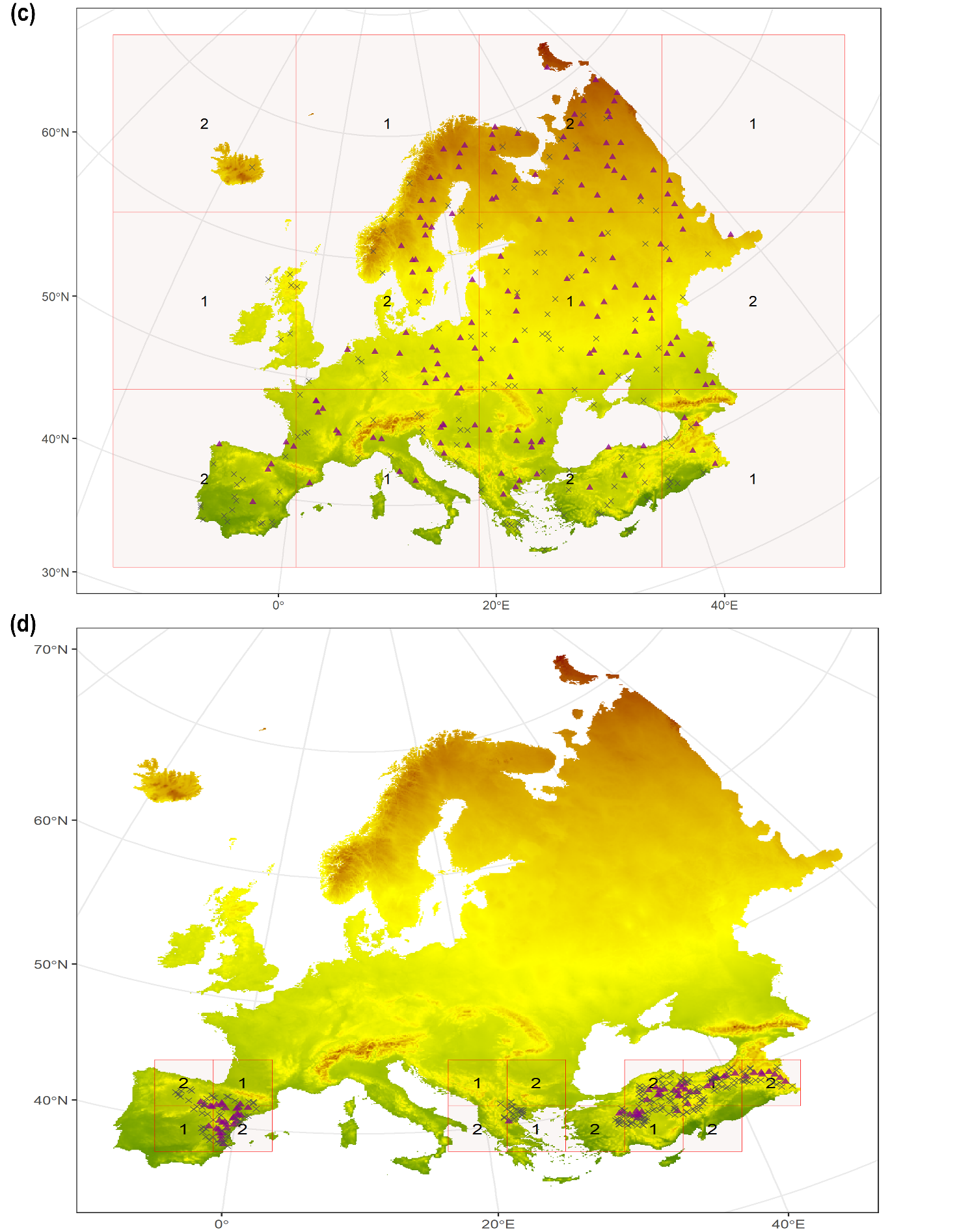
**Fig. S7.** (continues from previous page)

**Fig. S8.** Boxplots showing Root Mean Squared Error (RMSE) on the spatially independent test data selected through checkerboard spatial blocking, computed from the HSMs fitted for each VS * Worldclim version * Grid resolution * Algorithm combination. Red dots represent outliers.


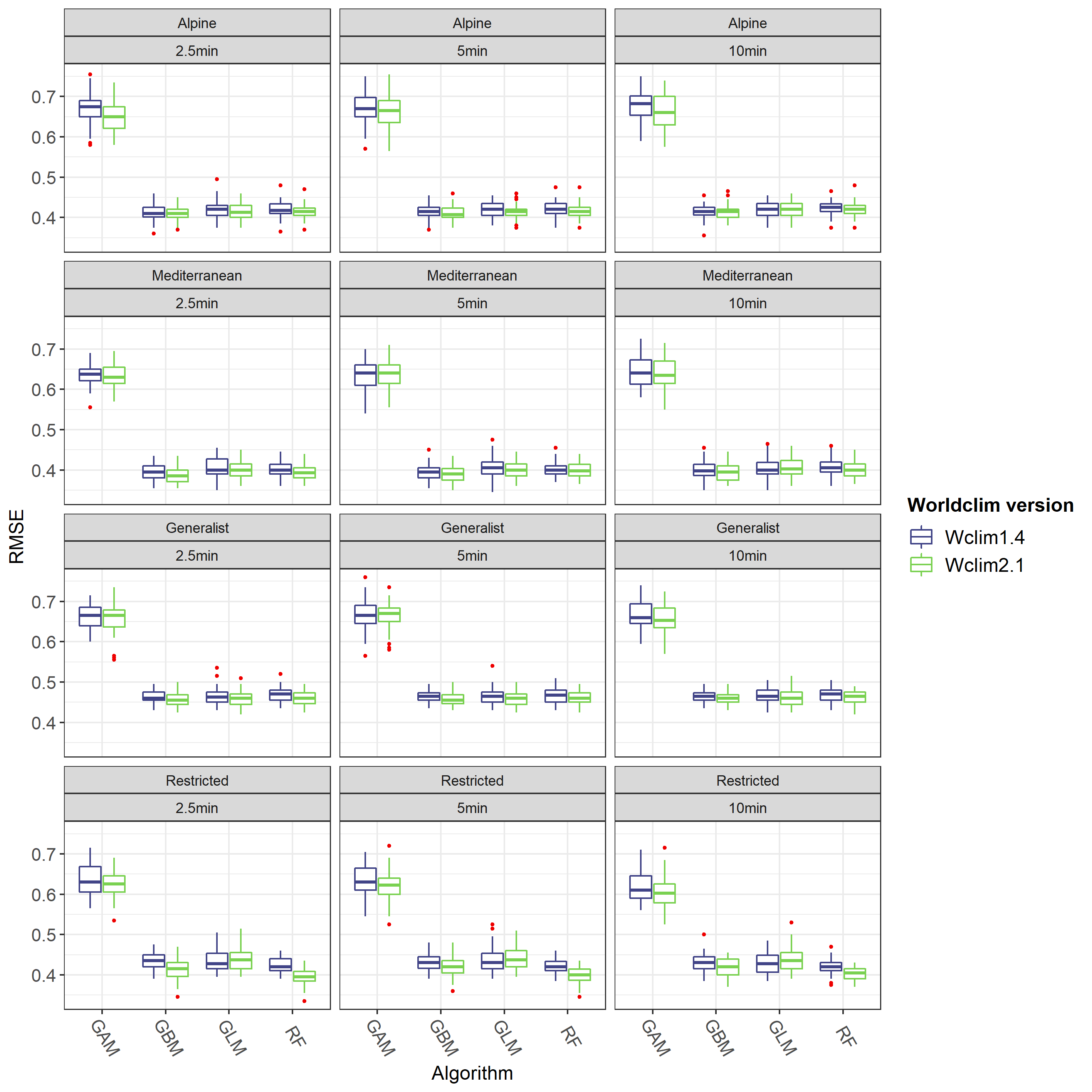


| **VS** | **Worldclim version** | **Grid resolution** | **EffectiveReps_Tot** | **HSMs_unique** | **EMs** |
| --- | --- | --- | --- | --- | --- |
| Alpine | Wclim1.4 | 2.5min | 50 | 0 | 50 |
| Alpine | Wclim2.1 | 2.5min | 50 | 0 | 50 |
| Alpine | Wclim1.4 | 5min | 50 | 0 | 50 |
| Alpine | Wclim2.1 | 5min | 50 | 0 | 50 |
| Alpine | Wclim1.4 | 10min | 50 | 0 | 50 |
| Alpine | Wclim2.1 | 10min | 50 | 0 | 50 |
| Mediterranean | Wclim1.4 | 2.5min | 50 | 0 | 50 |
| Mediterranean | Wclim2.1 | 2.5min | 50 | 0 | 50 |
| Mediterranean | Wclim1.4 | 5min | 50 | 0 | 50 |
| Mediterranean | Wclim2.1 | 5min | 50 | 0 | 50 |
| Mediterranean | Wclim1.4 | 10min | 50 | 0 | 50 |
| Mediterranean | Wclim2.1 | 10min | 50 | 0 | 50 |
| Generalist | Wclim1.4 | 2.5min | 23 | 8 | 15 |
| Generalist | Wclim2.1 | 2.5min | 32 | 10 | 22 |
| Generalist | Wclim1.4 | 5min | 26 | 10 | 16 |
| Generalist | Wclim2.1 | 5min | 29 | 6 | 23 |
| Generalist | Wclim1.4 | 10min | 23 | 8 | 15 |
| Generalist | Wclim2.1 | 10min | 25 | 9 | 16 |
| Restricted | Wclim1.4 | 2.5min | 39 | 17 | 22 |
| Restricted | Wclim2.1 | 2.5min | 48 | 5 | 43 |
| Restricted | Wclim1.4 | 5min | 35 | 10 | 25 |
| Restricted | Wclim2.1 | 5min | 47 | 9 | 38 |
| Restricted | Wclim1.4 | 10min | 39 | 14 | 25 |
| Restricted | Wclim2.1 | 10min | 43 | 9 | 34 |

**Table S1.** For each VS * Worldclim version * Grid resolution combination: number of sampling replicates for which at least one HSM exceeded the selected AUC and TSS thresholds (EffectiveReps_Tot); number of sampling replicates for which only one HSM exceeded the thresholds (HSMs_unique); number of sampling replicates for which at least two HSMs exceeded the thresholds, so that ensemble models could be built (EMs).

**Table S2.** For each VS * Grid resolution combination: correlation across Europe between the median HS computed from projections of Wclim2.1-based HSMs and the median HS computed from projections of Wclim1.4-based HSMs (Between-version *r*); correlation across Europe between the median HS computed from projections of Wclim1.4-based HSMs and the simulated occurrence probability (Wclim1.4-SimulatedProb. *r*); correlation across Europe between the median HS computed from projections of Wclim2.1-based HSMs and the simulated occurrence probability (Wclim2.1-SimulatedProb. *r*).

|  | **Alpine** | | |
| --- | --- | --- | --- |
|  | 2.5min | 5min | 10min |
| Between-version *r* | 0.99 | 0.99 | 0.99 |
| Wclim1.4-SimulatedProb. *r* | 0.98 | 0.98 | 0.98 |
| Wclim2.1-SimulatedProb. *r* | 0.98 | 0.98 | 0.98 |
|  | **Mediterranean** | | |
|  | 2.5min | 5min | 10min |
| Between-version *r* | 0.99 | 0.99 | 0.99 |
| Wclim1.4-SimulatedProb. *r* | 0.96 | 0.96 | 0.97 |
| Wclim2.1-SimulatedProb. *r* | 0.98 | 0.98 | 0.98 |
|  | **Generalist** | | |
|  | 2.5min | 5min | 10min |
| Between-version *r* | 0.96 | 0.96 | 0.96 |
| Wclim1.4-SimulatedProb. *r* | 0.89 | 0.9 | 0.9 |
| Wclim2.1-SimulatedProb. *r* | 0.93 | 0.94 | 0.94 |
|  | **Restricted** | | |
|  | 2.5min | 5min | 10min |
| Between-version *r* | 0.83 | 0.82 | 0.84 |
| Wclim1.4-SimulatedProb. *r* | 0.01 | 0 | 0.01 |
| Wclim2.1-SimulatedProb. *r* | 0.33 | 0.3 | 0.3 |


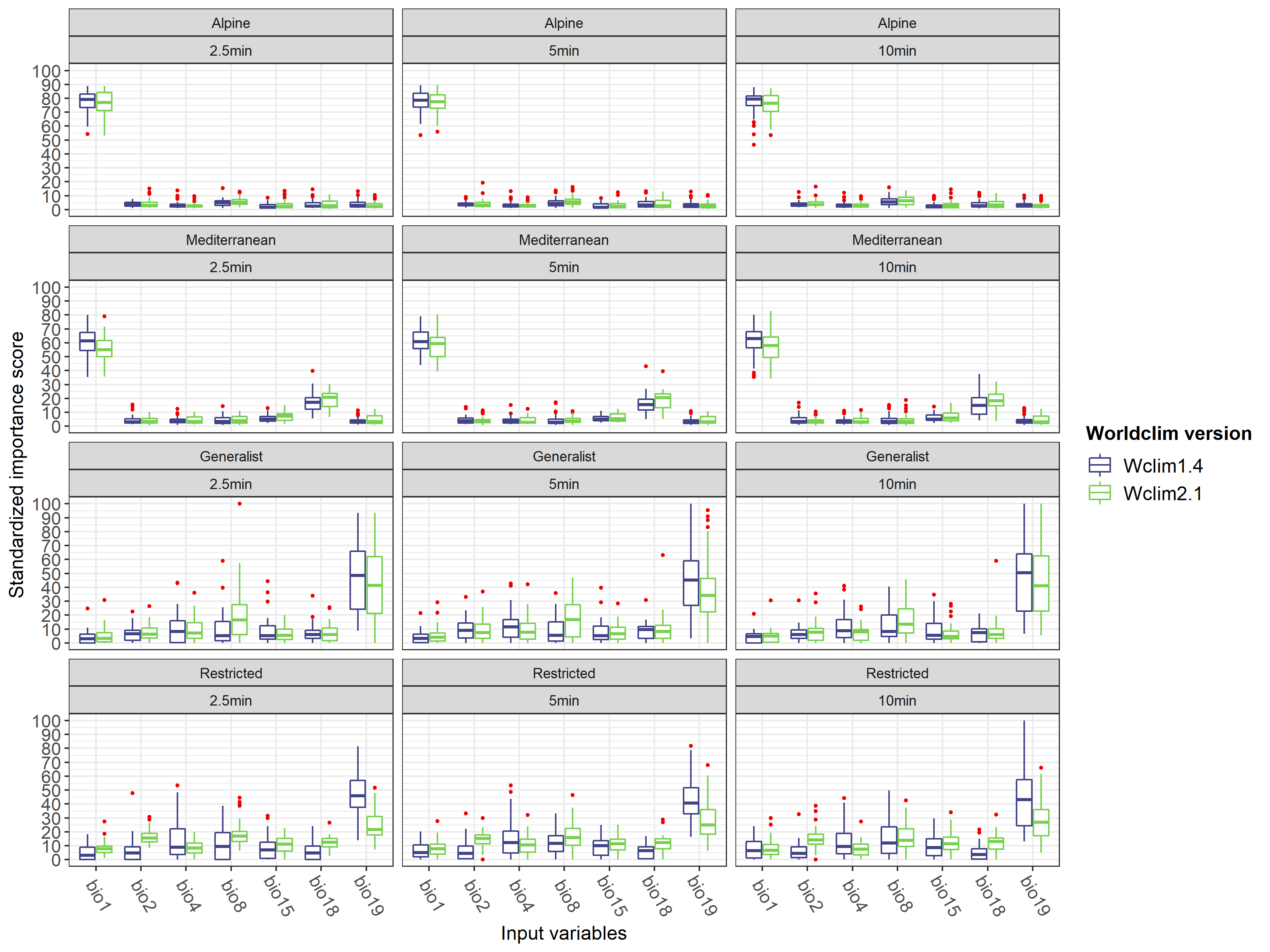
**Fig. S9.** Boxplots showing standardized importance scores computed, through the permutation-based (*n=3*) procedure implemented in ‘biomod2’, for the selected input variables from the HSMs exceeding the chosen AUC and TSS thresholds for each VS * Worldclim version * Grid resolution combination. Red dots represent outliers.
